# Group 2 Innate Lymphoid Cells Protect Mice from Abdominal Aortic Aneurysm Formation via IL5 and Eosinophils

**DOI:** 10.1002/advs.202206958

**Published:** 2023-01-02

**Authors:** Yuanyuan Zhang, Tianxiao Liu, Zhiyong Deng, Wenqian Fang, Xian Zhang, Shuya Zhang, Minjie Wang, Songyuan Luo, Zhaojie Meng, Jing Liu, Galina K. Sukhova, Dazhu Li, Andrew N. J. McKenzie, Peter Libby, Guo‐Ping Shi, Junli Guo

**Affiliations:** ^1^ Hainan Provincial Key Laboratory for Tropical Cardiovascular Diseases Research, Key Laboratory of Emergency and Trauma of Ministry of Education Institute of Cardiovascular Research of the First Affiliated Hospital Hainan Medical University Haikou 571199 China; ^2^ Department of Medicine Brigham and Women's Hospital and Harvard Medical School Boston MA 02115 USA; ^3^ Guangdong Provincial Geriatrics Institute Guangdong Provincial People's Hospital Guangdong Academy of Medical Sciences Guangzhou 510080 China; ^4^ Department of Geriatrics National Key Clinic Specialty Guangzhou First People's Hospital School of Medicine South China University of Technology Guangzhou 510180 China; ^5^ Cardiac Regeneration and Ageing Lab Institute of Cardiovascular Sciences School of Life Science Shanghai University Shanghai 200444 China; ^6^ Department of Cardiology Union Hospital Tongji Medical College Huazhong University of Science and Technology Wuhan 430022 China; ^7^ Division of Protein & Nucleic Acid Chemistry MRC Laboratory of Molecular Biology Cambridge CB2 0QH UK

**Keywords:** abdominal aortic aneurysm, eosinophil, Group‐2 innate lymphoid cell, IL5

## Abstract

Development of abdominal aortic aneurysms (AAA) enhances lesion group‐2 innate lymphoid cell (ILC2) accumulation and blood IL5. ILC2 deficiency in *Rora^fl/fl^Il7r^Cre/+^* mice or induced ILC2 depletion in *Icos^fl‐DTR‐fl/+^Cd4^Cre/+^* mice expedites AAA growth, increases lesion inflammation, but leads to systemic IL5 and eosinophil (EOS) deficiency. Mechanistic studies show that ILC2 protect mice from AAA formation via IL5 and EOS. IL5 or ILC2 from wild‐type (WT) mice, but not ILC2 from *Il5^−/−^* mice induces EOS differentiation in bone‐marrow cells from *Rora^fl/fl^Il7r^Cre/+^* mice. IL5, IL13, and EOS or ILC2 from WT mice, but not ILC2 from *Il5^−/−^* and *Il13^−/−^* mice block SMC apoptosis and promote SMC proliferation. EOS but not ILC2 from WT or *Il5^−/−^* mice block endothelial cell (EC) adhesion molecule expression, angiogenesis, dendritic cell differentiation, and Ly6C^hi^ monocyte polarization. Reconstitution of WT EOS and ILC2 but not *Il5^−/−^* ILC2 slows AAA growth in *Rora^fl/fl^Il7r^Cre/+^* mice by increasing systemic EOS. Besides regulating SMC pathobiology, ILC2 play an indirect role in AAA protection via the IL5 and EOS mechanism.

## Graphical abstract

The group‐2 innate lymphoid cells (ILC2) slow abdominal aortic aneurysm (AAA) development by producing IL5 and IL13 to block smooth muscle cell (SMC) apoptosis and promote SMC proliferation. ILC2 also produce IL5 to promote eosinophil differentiation as an indirect mechanism to affect SMC apoptosis and proliferation, and to block endothelial cell adhesion molecule expression, angiogenesis, dendritic cell differentiation, and Ly6C^hi^ monocyte polarization.

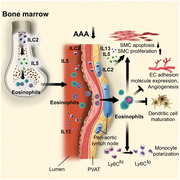

## 1 Introduction

Abdominal aortic aneurysm (AAA) is an inflammatory disease, characterized by the expansion of the abdominal aorta. Many aneurysms are small and asymptomatic, but have the potential to enlarge with risk of rupture. The current treatment of large AAA requires invasive open or endovascular surgical repairs.^[^ ^1^ ^]^ Despite improvements in interventional care, AAA patients have considerable residual morbidity and mortality risk. Therefore, greater understanding of the pathophysiology of AAA is needed to develop effective medical treatment and preventive strategies. The main mechanisms of AAA formation include aortic wall extracellular matrix degradation associated with aortic smooth muscle cell (SMC) loss, and inflammatory cell activation.^[^ ^2^, ^3^, ^4^ ^]^ Aortic inflammation contributes importantly to all stages of AAA development and substantially influences aortic wall remodeling. Innate and adaptive immune cells, such as neutrophils, macrophages, monocytes, dendritic cells (DCs), T cells, and B cells, all localize in aneurysms.^[^ ^5^, ^6^ ^]^ We have recently demonstrated the involvement of mast cells and eosinophils (EOS) in AAA. Mast cells promote AAA expansion by releasing cytokines (IL6, interferon‐*γ* [IFN‐*γ*]) and proteases (chymase, tryptase) to activate vascular and inflammatory cells.^[^ ^7^, ^8^, ^9^, ^10^ ^]^ In contrast, EOS slow AAA growth by releasing IL4 and cationic proteins to block matrix degradation, angiogenesis, and vascular and inflammatory cell activation, and promoting SMC proliferation and M2 macrophage and Ly6C^lo^ monocyte polarization.^[^ ^11^ ^]^

Group 2 innate lymphoid cells (ILC2) are newly identified innate source of type‐2 effector cytokines IL5 and IL13.^[^ ^12^, ^13^ ^]^ This group of cells are defined by lack of expression of myeloid or lymphoid lineage markers (CD11b, CD11c, Gr‐1, CD3) but expression of CD127, KLRG1(killer cell lectin‐like receptor G‐1), ST2, CD25, IL17BR, and GATA3. Expression of ST2, CD25, IL17BR, and CD127 allows ILC2 respond to their corresponding ligands, IL33, IL2, IL25, and TSLP (thymic stromal lymphopoietin). ILC2 populate epithelial barriers and adipose tissue, and exert well‐defined functions in adipose tissue, the airway, and the gastrointestinal tract^[^ ^14^, ^15^, ^16^ ^]^ as important regulators of innate and adaptive immune processes in metabolic diseases, asthma and allergic responses, and lung fibrosis.^[^ ^13^, ^14^, ^15^, ^16^, ^17^ ^]^ Yet, limited information is available regarding ILC2 function in cardiovascular disease. Indirect evidence showed that IL33, IL25, and TSLP increased the content of ILC2 in aortic lesions or the spleen, raised plasma ILC2 cytokines (IL5, IL13), and reduced murine atherosclerosis.^[^ ^18^, ^19^, ^20^, ^21^ ^]^ In mice fed a high‐fat diet, Lin^−^ICOS^+^CD25^+^CD127^+^KLRG1^+^ST2^+^CD90^+^Sca‐1^+^cKit^+^ ILC2 in peri‐aortic adipose tissue expressed high levels of IL5 and IL13 and triggered B1a cell proliferation and IgM natural antibody production.^[^ ^12^, ^22^ ^]^ A recent study showed that adoptive transfer of IL25‐induced splenic and lung ILC2 followed by in vitro expansion under IL7 and IL33 into apolipoprotein E‐deficient (*Apoe^−/−^*) mice reduced subvalvular heart and brachiocephalic artery lipid content, along with increased peritoneal B1 cells, EOS, and M2 macrophages. Yet, donor ILC2 did not change atherosclerotic lesion area,^[^ ^23^ ^]^ and donor ILC2 lost their surface expression of ST2 and KLRG1 after in vitro expansion.^[^ ^23^, ^24^ ^]^ It was not tested whether such changes in the phenotype of donor ILC2 contributed to the unaffected atherogenesis in *Apoe^−/−^* recipient mice. Nor is it clear that these in vitro‐expanded ILC2 acted the same as freshly isolated ILC2 in atherogenesis. Therefore, due to the lack of direct evidence, it remains unsettled whether ILC2 play any role in cardiovascular diseases.

This study defines a novel protective role of ILC2 in mouse AAA. ILC2 deficiency in *Rora^fl/fl^Il7r^Cre/+^* mice or diphtheria toxin (DTx)‐induced depletion of ILC2 in Icos^fl‐DTR‐fl/+^Cd4^Cre/+^ (*ICOS‐T*) mice expedited AAA expansion. Mechanistic studies demonstrated that ILC2 release IL5, IL13, and possibly other untested molecules to block SMC apoptosis and promote SMC proliferation. ILC2 also indirectly affect the functions of SMCs, endothelial cells (ECs) and other tested immune cells by releasing IL5 to promote EOS development.

## 2 Results

### 2.1 AAA Formation Increases ILC2 Accumulation in AAA Lesions and PaLN

**Figure 1:**
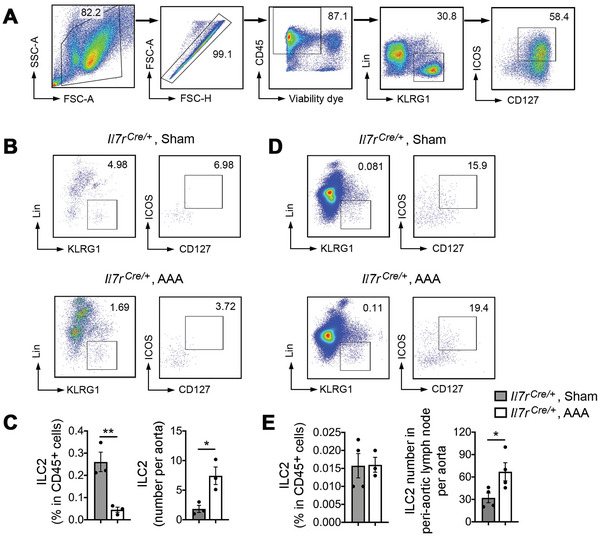
ILC2 accumulation in mouse CaPO_4_ injury‐induced AAA lesions and PaLN. A) Establishment of ILC2 gating strategy with splenocytes. Splenocytes were enriched using a lineage cell depletion kit. B) Representative FACS images of ILC2 from sham and CaPO_4_ injury‐induced AAA lesion from *Il7r^Cre^* mice. C) FACS determined lesion CD45^+^Lin^−^KLRG1^+^CD127^+^ICOS^+^ ILC2 percentage in live CD45^+^ cells and ILC2 absolute number per AAA lesion. D) Representative FACS images of PaLN ILC2 from *Il7r^Cre^* sham mice and *Il7r^Cre^* AAA mice. E) FACS determined PaLN CD45^+^Lin^−^KLRG1^+^CD127^+^ICOS^+^ ILC2 percentage in live CD45^+^ cells and ILC2 absolute number per AAA lesion. Data are mean±SEM, *n* = 3–4 per group, *p < 0.05, **p < 0.01, Mann‐Whitney *U* test.

To test ILC2 function in AAA, we produced peri‐aortic CaPO_4_‐induced AAA in *Il7r^Cre/+^* wild‐type (WT) mice. After 7 d, all mice developed AAA.^[^ ^25^ ^]^ To detect ILCs in the abdominal aorta or AAA lesions, we used FACS that allows detection of defined ILC2 surface markers. We first used the lineage cell depletion kit to enrich Lin^−^ cells in splenocytes from WT mice and established the gating strategy for CD45^+^Lin^−^KLRG1^+^CD127^+^ICOS^+^ ILC2 (**Figure** **1**A). FACS analysis demonstrated increased ILC2 in AAA lesions, although the ILC2 percentage among total CD45^+^ cells was significantly lower in AAA lesions, likely because of the known increase of total inflammatory cells in AAA lesions (Figure 1B/C). Due to the small numbers of ILC2 in each AAA fragment, we combined three aortas or AAA lesion fragments for each FACS analysis. All cells from AAA lesion single cell preparation were combined for analysis. Prior studies showed that the peri‐vascular adipose tissue and peri‐aortic lymph node (PaLN) contain ILC2.^[^ ^26^ ^]^ Development of AAA did not change the ILC2 percentage among CD45^+^ cells, but increased the absolute number of ILC2 in the PaLN (Figure 1D/E).

### 2.2 ILC2 Deficiency Aggravates Mouse AAA Growth and Lesion Inflammation

**Figure 2:**
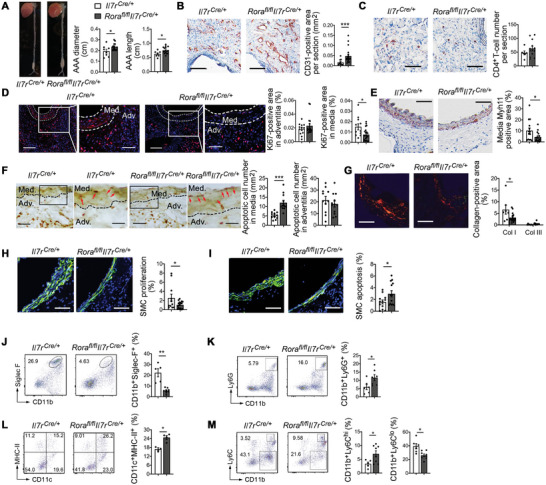
ILC2 ablation aggravates CaPO_4_ injury‐induced AAA growth and lesion inflammation. A) Abdominal aortic diameter, length, and representative images of AAA from *Il7r^Cre/+^* WT and ILC2‐deficient *Rora^fl/fl^Il7r^Cre/+^* mice. Powers: 0.5360 and 0.66. B) Lesion CD31^+^ microvessel areas. C) Lesion CD4^+^ T‐cell contents. D) Lesion Ki67^+^ proliferating cells in media and adventitia. E) Lesion Myh11‐positive SMC areas. F) Lesion media and adventitia TUNEL‐positive apoptotic cell contents. G) Sirius red staining. H) Immunofluorescent staining of *α*‐actin (green) and Ki67 (red) to detect lesion SMC proliferation. I) Immunofluorescent staining of *α*‐actin (green) and cleaved caspase‐3 (red) to detect lesion SMC apoptosis. FACS analysis of J) AAA lesion CD11b^+^Siglec‐F^+^ EOS, K) CD11b^+^Ly6G^+^ neutrophils, L) CD11c^+^MHC‐II^+^ DCs, and M) CD11b^+^Ly6C^hi^ and CD11b^+^Ly6C^lo^ monocytes from *Il7r^Cre/+^* and *Rora^fl/fl^Il7r^Cre/+^* mice. Representative images in panels (A) to (I) are shown to the left. Scales: 100 µm; inset scales in (D) and (F): 50 µm. Data are mean±SEM, *n* = 8 and 15, *p < 0.05, **p < 0.01, ***p < 0.001, nonparametric Mann–Whitney *U* test.

Although ILC2 are of relatively low frequency (≈0.1% of total lymphocytes), they play essential roles in obesity, parasite infection, allergic inflammation, and arthritis.^[^ ^13^, ^14^, ^15^, ^16^, ^17^ ^]^ To test the participation of ILC2 in AAA, we performed peri‐aortic CaPO_4_ injury and induced AAA in both ILC2‐deficient (retinoid‐related orphan receptor alpha) *Rora^fl/fl^Il7r^Cre/+^* and *Il7r^Cre/+^* WT control mice as we previously reported.^[^ ^25^ ^]^ Earlier studies indicated a role for ILC2‐derived IL5 in EOS development.^[^ ^27^ ^]^ *Rora* is not restricted only to the ILC2, but also expressed in regulatory T cells (Treg) and T helper (Th) cells.^[^ ^28^, ^29^, ^30^ ^]^ These immune cells may be affected in *Rora^fl/fl^Il7r^Cre/+^* mice. The original study reported ILC2 deficiency in mesenteric lymph nodes from *Rora^fl/fl^Il7r^Cre/+^* mice, but CD4^+^ T cells and NK cells were not affected.^[^ ^31^ ^]^ To confirm the deficiency of ILC2 but no other immune cells in *Rora^fl/fl^Il7r^Cre/+^* mice, we performed FACS analysis of Lin^−^KLRG1^+^ICOS^+^ CD127^+^ ILC2, CD11b^+^Siglac‐F^+^ EOS, CD3^+^CD4^+^CD25^+^Foxp3^+^ Treg, CD3^+^CD4^+^IFN‐*γ* ^+^ Th1 cells, CD3^+^CD4^+^IL4^+^ Th2 cells, and CD3^+^CD4^+^IL17^+^ Th17 cells in blood, spleen, bone‐marrow, and abdominal aortas from *Rora^fl/fl^Il7r^Cre/+^* and *Il7r^Cre/+^* control mice. The results showed that both ILC2 and EOS, but not Treg, Th1, Th2, and Th17 cells were deficient in blood, spleen, and bone‐marrow from *Rora^fl/fl^Il7r^Cre/+^* mice, whereas all these tested cells were negligible in normal mouse aortas (Figures S1–S6, Supporting Information). At 7 d after CaPO_4_ injury‐induced AAA, we detected significantly larger lesion sizes (both length and diameter) in *Rora^fl/fl^Il7r^Cre/+^* mice than those in *Il7r^Cre/+^* mice (**Figure** **2**A). Compared with those in *Il7r^Cre^* mice, AAA lesions from *Rora^fl/fl^Il7r^Cre/+^* mice contained significantly more CD31^+^ microvessels, although lesion CD4^+^ T‐cell content did not differ (**Figure** **2**B/C). ILC2 deficiency did not affect lesion adventitial cell proliferation or apoptosis, but significantly reduced medial cell proliferation and augmented medial cell apoptosis and SMC loss (Figure 2D–F). Consistent with these observations, Sirius red staining revealed loss of type‐I collagen (red‐orange color) in AAA lesions from the *Rora^fl/fl^Il7r^Cre/+^* mice (Figure 2G). Immunofluorescent double staining showed reduced lesion Ki67^+^ *α*‐actin^+^ double‐positive proliferating SMCs but increased caspase‐3^+^ *α*‐actin^+^ apoptotic SMCs in AAA lesions from *Rora^fl/fl^Il7r^Cre/+^* mice (Figure 2H/I), suggesting that the loss of medial SMCs in these mice results from less SMC proliferation and increased SMC apoptosis. Besides these morphological changes, we found that ILC2 deficiency in *Rora^fl/fl^Il7r^Cre/+^* mice led to reduced CD11b^+^Siglec‐F^+^ EOS in AAA lesions (Figure 2J), and increased lesional CD11b^+^Ly6G^+^ neutrophils, CD11c^+^MHC‐II^+^ DCs, and CD11b^+^Ly6C^hi^ monocytes (Figure 2K–M). FACS analysis also showed reduction of CD11b^+^Ly6C^lo^ monocytes in AAA lesions from *Rora^fl/fl^Il7r^Cre/+^* mice (Figure 2M). Earlier studies demonstrated the participation of neutrophils in AAA.^[^ ^32^ ^]^ The maturation of DCs often follows activation of antigen‐presenting and pro‐inflammatory activity. Depletion of DCs can inhibit AAA growth.^[^ ^33^ ^]^ Although the exact role of different monocyte subsets in AAA remains incompletely understood, AAA lesions contain both CD11b^+^Ly6C^hi^ and CD11b^+^Ly6C^lo^ monocytes.^[^ ^34^ ^]^ We recently reported a protective role of EOS in angiotensin‐II (Ang‐II) infusion‐induced AAA in mice.^[^ ^11^ ^]^ Therefore, the increase of pro‐inflammatory neutrophils, DCs, and CD11b^+^Ly6C^hi^ monocytes, but decrease of anti‐inflammatory EOS and CD11b^+^Ly6C^lo^ monocytes, may result from increased AAA growth due to loss of ILC2. Alternatively, the altered population of inflammatory cells may contribute directly or indirectly to the exacerbated AAA growth in *Rora^fl/fl^Il7r^Cre/+^* mice.

Several AAA models have been broadly used, including Ang‐II infusion, aortic elastase perfusion, in addition to peri‐aortic CaCl_2_ injury, although none of them fully recapitulates human AAA development. Ang‐II infusion causes systemic inflammation in addition to blood pressure increase, elastase perfusion damages aortic wall elastica, and peri‐aortic CaCl_2_ injury promotes in situ matrix remodeling, SMC apoptosis, and inflammation.^[^ ^35^ ^]^ To test further a protective role for ILC2 in AAA development, we produced *Apoe^−/−^Rora^fl/fl^Il7r^Cre/+^* and *Apoe^−/−^Il7r^Cre/+^* control mice and performed Ang‐II subcutaneous perfusion. After 28 d, *Apoe^−/−^Rora^fl/fl^Il7r^Cre/+^* mice developed significantly larger and longer suprarenal AAA lesion size and higher aortic expansion rate and AAA incidences than the *Apoe^−/−^Il7r^Cre/+^* control mice (Figure S7A, Supporting Information). AAA lesions from *Apoe^−/−^Rora^fl/fl^Il7r^Cre/+^* mice also showed more media cell apoptosis, less lesion media Myh11‐positive SMC contents and lesion collagen‐I deposition than those of *Apoe^−/−^Il7r^Cre/+^* control mice (Figure S7B–D, Supporting Information), although both *Apoe^−/−^Rora^fl/fl^Il7r^Cre/+^* and *Apoe^−/−^Il7r^Cre/+^* control mice showed similar systolic, diastolic, and mean blood pressure increases after AAA development (Figure S7E, Supporting Information). Instead of intravascular elastase perfusion, we also produced peri‐aortic elastase injury‐induced AAA in *Rora^fl/fl^Il7r^Cre/+^* and *Il7r^Cre/+^* control mice as previously reported.^[^ ^36^, ^37^ ^]^ After 28 d, ILC2‐deficient *Rora^fl/fl^Il7r^Cre/+^* mice again developed significantly larger lesions and much higher percentages of aortic expansion than did the *Il7r^Cre/+^* control mice (Figure S8A/B, Supporting Information), although the increase of lesion length did not reach statistical significance (Figure S8C, Supporting Information). While only 30% of *Il7r^Cre/+^* control mice developed AAA as defined by 150% or greater aortic expansion, all *Rora^fl/fl^Il7r^Cre/+^* mice (100%) developed AAA (Figure S8D, Supporting Information).

### 2.3 ILC2 Deficiency Impairs IL5 Secretion and EOS Development

**Figure 3:**
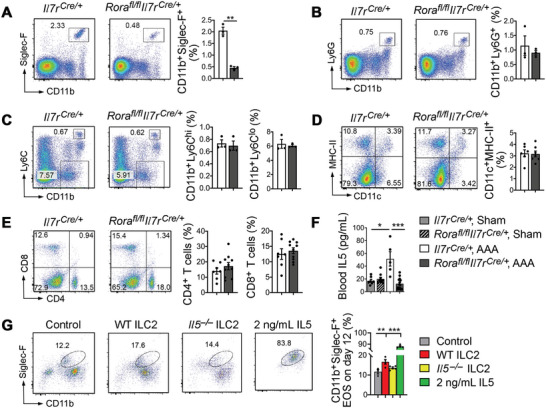
ILC2 deficiency blocks IL5‐dependent EOS generation. FACS analysis of A) splenic CD11b^+^Siglec‐F^+^ EOS, B) CD11b^+^Ly6G^+^ neutrophils, C) CD11b^+^Ly6C^hi^ and CD11b^+^Ly6C^lo^ monocytes, D) CD11c^+^MHC‐II^+^ DCs, and E) CD4^+^CD8^−^ and CD4^−^CD8^+^ T cells from *Il7r^Cre/+^* and *Rora^fl/fl^Il7r^Cre/+^* mice after peri‐aortic CaPO_4_‐induced AAA. F) Blood IL5 levels in *Il7r^Cre^* and *Rora^fl/fl^Il7r^Cre/+^* mice at 7 d after peri‐aortic sham or CaPO_4_ injury‐induced AAA. G) FACS analysis of CD11b^+^Siglec‐F^+^ EOS in bone‐marrow cells from *Rora^fl/fl^Il7r^Cre/+^* mice after 12 d of culture with or without ILC2 lysates from WT and *Il5^−/−^* mice, or 2 ng mL^−1^ of IL5 as a positive control. Data are mean±SEM. *n* = 3–16 per group. *p < 0.05, **p < 0.01, ***p < 0.001, A–E) nonparametric Mann–Whitney *U* test, or F,G) one‐way ANOVA test.

To test whether ILC2 deficiency in *Rora^fl/fl^Il7r^Cre/+^* mice also affected systemic inflammatory cell profiles after AAA formation, we assessed the same sets of immune cells in the spleens as in AAA lesions. Like in mice without AAA (Figure S2, Supporting Information), FACS analysis of splenocytes from *Il7r^Cre/+^* and *Rora^fl/fl^Il7r^Cre/+^* mice after CaPO_4_ injury‐induced AAA demonstrated that ILC2 deficiency reduced splenic CD11b^+^Siglec‐F^+^ EOS (**Figure** **3**A). Yet, ILC2 deficiency did not affect any other tested immune cells, including CD11b^+^Ly6G^+^ neutrophils, CD11b^+^Ly6C^hi^ and CD11b^+^Ly6C^lo^ monocytes, CD11c^+^MHC‐II^+^ DCs, and CD4^+^ or CD8^+^ T cells (Figure 3B–E). EOS deficiency in spleens from *Rora^fl/fl^Il7r^Cre/+^* mice with or without AAA suggests a congenital deficiency of IL5 in the bone‐marrow where IL5 controls EOS maturation.^[^ ^38^ ^]^ Mice without AAA displayed significant reduction of IL5 expression by immunoblot analysis in the bone‐marrow from *Rora^fl/fl^Il7r^Cre/+^* mice (Figure S9A, Supporting Information). Consistent with this result, we detected deficiency of EOS (Figure S2B/S9B, Supporting Information), but not neutrophils, monocytes, DCs, and CD4^+^ and CD8^+^ T cells in spleens from *Rora^fl/fl^Il7r^Cre/+^* mice without AAA (Figure S9C–F, Supporting Information). These observations suggest that ILC2 are essential to the development of EOS, independent of AAA formation. Peripheral blood from the *Il7r^Cre/+^* mice showed significant elevation of IL5 at 7 d after peri‐aortic CaPO_4_ exposure. ILC2 deficiency in *Rora^fl/fl^Il7r^Cre/+^* mice lowered blood IL5 (Figure 3F). To test participation of ILC2 in bone‐marrow EOS development, we cultured mouse bone‐marrow cells from *Rora^fl/fl^Il7r^Cre/+^* mice with exogenous ILC2 lysates from WT or *Il5^−/−^* mice. Using ELISA, we detected about 50 pg of IL5 from 2 × 10^5^ freshly isolated ILC2. Using FACS, we detected significant increase of CD11b^+^Siglec‐F^+^ EOS from bone‐marrow cell culture after 12 d of treatment with ILC2 lysates from WT mice. ILC2 lysates from *Il5^−/−^* mice showed a blunted response. Recombinant IL5 at 2 ng mL^−1^ served as a positive control (Figure 3G). These data support a vital role for ILC2 and ILC2‐derived IL5 in EOS generation.

### 2.4 ILC2 and EOS Protect SMCs from Apoptosis and Promote SMC Proliferation

**Figure 4:**
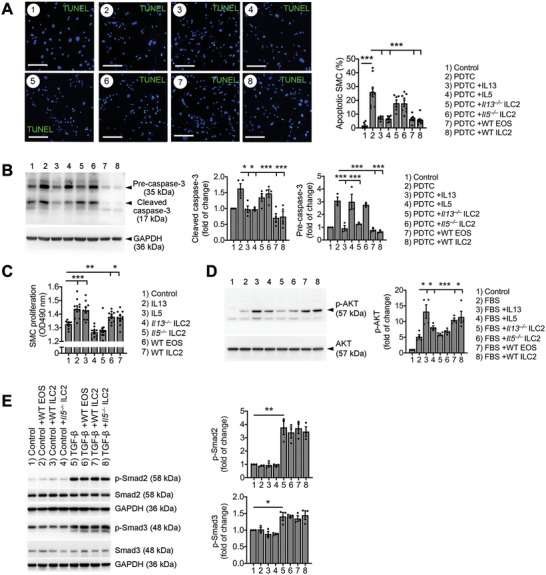
ILC2 and EOS interactions with SMCs. A) TUNEL staining detected PDTC‐induced aortic SMC apoptosis with or without lysates from WT EOS, WT ILC2, *Il5^−/−^* and *Il13^−/−^* ILC2, or recombinant IL5 and IL13 (10 ng mL^−1^). Scale: 50 µm. B) Immunoblot analysis detected pre‐caspase‐3 and cleaved caspase‐3 in PDTC‐induced aortic SMC apoptosis under different conditions as indicated. C) Aortic SMC proliferation under the treatments as indicated. D) Immunoblot analysis detected total AKT and p‐AKT in aortic SMCs treated with or without FBS and other conditions as indicated. E) Immunoblot analysis of TGF‐*β*‐induced p‐Smad2/3 signaling from aortic SMCs pretreated with or without EOS lysates from WT mice or ILC2 lysates from WT and *Il5^−/−^* mice. Representative images in panels (A), (B), (D), and (E) are shown to the left. The extra band in the p‐Smad3 blot after TGF‐*β* treatment remains unknown. Data are mean±SEM. *n* = 10 per group. *p < 0.05, **p < 0.01, ***p < 0.001, one‐way ANOVA test for normal distributed samples (after Shapiro–Wilk test), followed by LSD correction.

Increased media SMC loss and apoptosis, and decreased SMC proliferation and collagen deposition in AAA lesions from *Rora^fl/fl^Il7r^Cre/+^* mice (Figure 2D–I) suggest that ILC2 prevent aortic SMCs from apoptosis or promote SMC activation or proliferation. Results from Figure 3 and Figures S2 and S9 (Supporting Information) indicated an essential role for ILC2 and ILC2‐derived IL5 in bone‐marrow EOS development. We recently reported that EOS promote SMC proliferation and protect SMCs from NF‐*κ*B activation.^[^ ^11^ ^]^ Therefore, ILC2 preservation of SMCs from apoptosis or promotion of SMC activation or proliferation may involve both a direct action of ILC2‐derived cytokines on SMCs and an indirect role of ILC2 on SMCs via the IL5 and EOS mechanism. Testing of this hypothesis used pyrrolidine dithiocarbamate (PDTC) to induce apoptosis of mouse aortic SMCs prepared from WT mice. Cell lysate preparations from EOS, or ILC2 from WT mice, but not ILC2 from *Il5^−/−^* or *Il13^−/−^* mice, blocked PDTC‐induced SMC apoptosis. Mouse recombinant IL5 or IL13 at 10 ng mL^−1^ also protected SMCs from PDTC‐induced apoptosis as previously reported (**Figure** **4**A).^[^ ^39^ ^]^ Immunoblot analysis showed that PDTC‐induced production of cleaved caspase‐3 was blunted by recombinant IL5 and IL13 and ILC2 and EOS from WT mice but not ILC2 from *Il5^−/−^* and *Il13^−/−^* mice (Figure 4B). These results suggest that the enhanced SMC apoptosis in AAA lesions from *Rora^fl/fl^Il7r^Cre/+^* mice (Figure 2I) related to the absence of ILC2, deficiency of IL5 (Figure 3F), and a reduction of lesion and peripheral EOS (Figure 2J/3A and Figure S9B, Supporting Information).

AAA lesions from *Rora^fl/fl^Il7r^Cre/+^* mice also showed lower SMC proliferation as determined by *α*‐actin and Ki67 immunofluorescent double staining (Figure 2H), suggesting that ILC2 promote SMC proliferation. We tested this hypothesis by culturing mouse aortic SMCs with EOS and ILC2 lysates or recombinant IL5 and IL13. Use of EOS and ILC2 lysates from WT mice or recombinant IL5 and IL13 enhanced SMC proliferation. In contrast, ILC2 lysates from *Il5^−/−^* or *Il13^−/−^* mice failed to induce aortic SMC proliferation (Figure 4C). To explore further the role of ILC2 and their IL5 and IL13 in SMC activation, we stimulated SMCs with fetal bovine serum (FBS). FBS‐induced SMC p‐AKT signaling^[^ ^40^ ^]^ was increased further by IL5 and IL13 and by ILC2 and EOS from WT mice. Such activity was muted when ILC2 from *Il5^−/−^* and *Il13^−/−^* mice were used (Figure 4D). These observations suggest that ILC2 and ILC2‐derived IL5 and IL13 played a direct role in protecting SMCs from apoptosis and promoting SMC proliferation. These results also suggest that ILC2 protected SMCs from apoptosis and promoted SMC proliferation indirectly via the EOS.

AAA lesions from *Rora^fl/fl^Il7r^Cre/+^* mice also contained less collagen I than in those from *Il7r^Cre^* control mice (Figure 2G). Vascular SMCs produce the bulk of aortic collagens.^[^ ^41^ ^]^ To test whether ILC2, EOS, or IL5 affected mouse aortic SMC collagen expression, we cultured mouse aortic SMCs with or without EOS and ILC2 lysates from WT mice or ILC2 lysates from *Il5^−/−^* mice. With or without TGF‐*β*, EOS or ILC2 lysates from WT or *Il5^−/−^* mice did not affect p‐Smad2 or p‐Smad3 activation (Figure 4E). IL5 yielded similar results. Different concentrations of IL5 (10–200 ng mL^−1^) did not affect p‐Smad2/3 activation, as determined by immunoblot analyses (Figure S10A/B, Supporting Information).Therefore, reduced collagen deposition in AAA lesions from *Rora^fl/fl^Il7r^Cre/+^* mice (Figure 2G) may associate with increased loss of SMCs from these mice (Figure 2E) due to increased SMC apoptosis (Figure 2I) and reduced SMC proliferation (Figure 2H) rather than interference with p‐Smad2/3 signaling.

### 2.5 ILC2 Actions on ECs and Inflammatory Cells Rely on EOS

**Figure 5:**
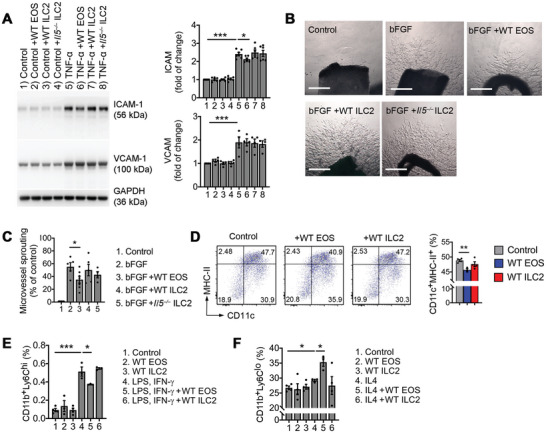
ILC2 and EOS interactions with ECs, DCs, and monocytes. A) Immunoblot detected ICAM‐1 and VCAM‐1 expression from aortic ECs pretreated with or without EOS lysates from WT mice or ILC2 lysates from WT or *Il5^−/−^* mice. B/C) Mouse aortic ring assay representative images and quantification. bFGF was used as positive controls. Scale: 400 µm. D) FACS analysis of bone‐marrow‐derived DCs cultured with or without EOS lysates and ILC2 lysates from WT mice. FACS analysis of CD11b^+^Ly6C^hi^ monocytes after bone‐marrow‐derived monocytes were treated with E) LPS and INF‐*γ*, and F) CD11b^+^Ly6C^lo^ monocytes after cells were treated with IL4, with and without EOS lysates and ILC2 lysates from WT mice. Representative images in panels (A) and (D) are shown to the left. Data are mean±SEM. *n* = 3–7 per group. *p < 0.05, **p < 0.01, ***p < 0.001, one‐way ANOVA test.

In distinction from SMCs, other AAA lesion cells, including ECs, DCs, and monocytes showed different responses to ILC2. With or without TNF‐*α* activation, ILC2 lysates from WT or *Il5^−/−^* mice did not affect EC expression of adhesion molecules ICAM‐1 (intercellular adhesion molecule‐1) or VCAM‐1 (vascular cell adhesion molecule‐1). Only EOS lysates from WT mice modestly reduced aortic EC expression of ICAM‐1, although such reduction reached statistical significance as determined by immunoblot analyses (**Figure** **5**A). An aortic ring angiogenesis assay yielded similar results. Only EOS lysates from WT mice, but not ILC2 lysates from WT or *Il5^−/−^* mice reduced bFGF‐induced microvessel growth (Figure 5B/C, Supporting Information). EOS lysates but not ILC2 lysates from WT mice reduced DC differentiation from WT bone‐marrow cells (Figure 5D). This finding may not explain increased DCs in AAA lesions from *Rora^fl/fl^Il7r^Cre/+^* mice (Figure 2L) because splenic DCs in the same *Rora^fl/fl^Il7r^Cre/+^* mice did not differ from those in *Il7r^Cre/+^* control mice after AAA development (Figure 3D). Increased DCs in AAA lesions from *Rora^fl/fl^Il7r^Cre/+^* mice may result from their increased AAA growth. Monocytes polarize to Ly6C^hi^ phenotypes in the presence of lipopolysaccharide (LPS) and IFN‐*γ*, while IL4 promotes Ly6C^lo^ monocyte polarization.^[^ ^42^, ^43^ ^]^ When bone‐marrow‐derived monocytes were treated with LPS and IFN‐*γ*, EOS—but not ILC2 from WT mice—blocked Ly6C^hi^ monocyte generation (Figure 5E). Similarly, when bone‐marrow‐derived monocytes were treated with IL4, EOS—but not ILC2 from WT mice—increased Ly6C^lo^ monocyte generation (Figure 5F).

### 2.6 ILC2 and EOS Attenuate AAA Growth in ILC2‐deficient Mice

**Figure 6:**
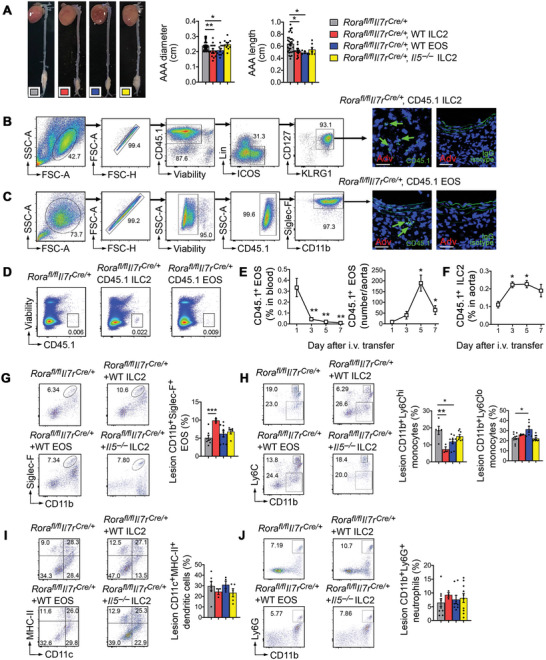
Reconstitution of ILC2 or EOS reduces AAA growth in ILC2‐deficient *Rora^fl/fl^Il7r^Cre/+^* mice. A) Aortic diameter and length, and representative images of *Rora^fl/fl^Il7r^Cre/+^* mice treated with or without WT EOS or ILC2 from WT and *Il5^−/−^* mice. Power: 1.000. B) ILC2 sorting strategy from WT splenocytes. ILC2 were defined as CD45^+^Lin^−^ ICOS^+^CD127^+^KLRG1^+^ cells and isolated from CD45.1 transgenic mice. Donor CD45.1^+^ ILC2 cells were used to monitor their accumulation in *Rora^fl/fl^Il7r^Cre/+^* mouse AAA lesions by CD45.1 immunofluorescent staining. C) FACS demonstrated the purity of bone‐marrow‐derived CD11b^+^Siglec‐F^+^ EOS after 12 d differentiation. Donor CD45.1^+^ EOS were also detected in *Rora^fl/fl^Il7r^Cre/+^* mouse AAA lesions using CD45.1 immunofluorescent staining. D) FACS analysis of blood donor CD45.1^+^ ILC2 or EOS in *Rora^fl/fl^Il7r^Cre/+^* mice at 7 d after reconstitution and peri‐aortic CaPO_4_ injury. E/F) FACS analysis of donor CD45.1^+^ EOS in blood and CD45.1^+^ ILC2 in aorta from *Rora^fl/fl^Il7r^Cre/+^* recipient mice at different days (1, 3, 5, 7) after donor cell adoptive transfer. FACS analysis of G) CD11b^+^Siglec‐F^+^ EOS, H) CD11b^+^Ly6C^hi^ and CD11b^+^Ly6C^lo^ monocytes, I) CD11c^+^MHC‐II^+^ DCs, and J) CD11b^+^Ly6G^+^ neutrophils in AAA lesions from *Rora^fl/fl^Il7r^Cre/+^* mice with or without receiving donor EOS or ILC2 from WT and *Il5^−/−^* mice. Data are mean±SEM, *n* = 10–25 per group, *p < 0.05, **p < 0.01, ***p < 0.001, one‐way ANOVA test for normal distributed samples (after Shapiro–Wilk test) followed by A,F–I) LSD correction or E) nonparametric Mann–Whitney *U* test.

Our in vitro studies demonstrated that ILC2‐derived IL5 and IL13 and EOS protected SMCs from apoptosis and promoted SMC proliferation (Figure 4A–D). Only EOS, but not ILC2 blocked EC adhesion molecule expression and angiogenesis, DC differentiation, and Ly6C^lo^ to Ly6C^hi^ monocyte polarization (Figure 5A–F) and depended on IL5 from ILC2 (Figure 3G). These observations suggest that ILC2 blocked AAA growth directly by acting on lesional SMCs and indirectly by acting on SMCs, ECs, and lesion inflammatory cells via the IL5 and EOS mechanisms. To test these possibilities, we performed adoptive transfer of ILC2 and EOS to ILC2‐deficient *Rora^fl/fl^Il7r^Cre/+^* mice. Donor ILC2 and EOS from WT mice, but not ILC2 from *Il5^−/−^* mice, reduced AAA diameter and length in *Rora^fl/fl^Il7r^Cre/+^* recipient mice (**Figure** **6**A). Donor EOS were prepared from bone‐marrow cells as we recently reported.^[^ ^11^, ^44^ ^]^ Due to the relatively low frequency of ILC2 (≈0.1% of total lymphocytes) and their potential surface marker alterations from in vitro expansion,^[^ ^23^, ^24^ ^]^ we used freshly prepared ILC2 from mouse splenocytes after donor mice were treated with IL33 to boost ILC2 expansion in vivo.^[^ ^45^ ^]^ Donor ILC2 (CD45.1^+^Lin^−^ICOS^+^CD127^+^KLRG1^+^) and EOS (CD45.1^+^CD11b^+^Siglec‐F^+^) from CD45.1 transgenic mice were used to confirm their homing to the AAA lesions, 7 d after surgery, by immunofluorescent staining with the CD45.1 antibody. IgG isotypes were used as experimental controls (Figure 6B/C). Earlier studies used in vitro expanded ILC2 as donor cells for both in vitro and in vivo tests, but those ILC2 lost their surface expression of ST2 and KLRG1 after in vitro expansion.^[^ ^23^, ^24^ ^]^ Instead, we used splenic ILC2 directly from IL33‐treated mice. Using donor ILC2 from CD45.1 transgenic mice, we performed immunofluorescent staining and detected the expression of ST2 and ICOS from CD45.1^+^ donor ILC2 in *Rora^fl/fl^Il7r^Cre/+^* recipient mouse AAA lesions (Figure S11A/B, Supporting Information). Donor ILC2 in AAA lesions also expressed CD25, programmed cell death protein‐1 (PD1), IL5, and IL13 (Figure S12A–D, Supporting Information). As we reported recently,^[^ ^11^, ^44^ ^]^ CD45.1^+^ donor EOS decreased to the baseline in blood at 7 d after adoptive transfer, but CD45.1^+^ donor ILC2 remained high in the blood (Figure 6D/E, left). In AAA lesions, the absolute number of CD45.1^+^ donor EOS peaked at 5 d and dropped by 75% at 7 d after adoptive transfer (Figure 6E, right), but the percentage of donor ILC2 in AAA lesions remained high (Figure 6F). This observation is consistent to the long lifespan of ILC2.^[^ ^27^, ^46^ ^]^

Adoptive transfer of ILC2 from WT mice, but not from *Il5^−/−^* mice, increased AAA lesion CD11b^+^Siglec‐F^+^ EOS and reduced lesion CD11b^+^Ly6C^hi^ monocytes, but did not affect AAA lesion DCs or neutrophils (Figure 6G–J). In contrast, donor EOS did not increase lesion EOS content (Figure 6G). This result resembles what we saw in recipient mouse hearts. At 3 d after adoptive transfer, donor EOS in recipient mouse hearts dropped by ≈60%.^[^ ^44^ ^]^ The reduction of EOS in *Rora^fl/fl^Il7r^Cre/+^* recipient mouse AAA lesions at 7 d after AAA production (Figure 6G) agreed with the sharp reduction of lesion donor EOS at this time point (Figure 6E). High lesional EOS content in ILC2‐treated *Rora^fl/fl^Il7r^Cre/+^* recipient mice may associate with high donor ILC2 in the bone‐marrow where ILC2 controls EOS development. FACS analysis revealed high content of donor CD45.1^+^ ILC2 in recipient mouse bone‐marrow at all tested time points after adoptive transfer and AAA production (Figure S13A/B, Supporting Information). Donor EOS reduced AAA lesion CD11b^+^Ly6C^hi^ monocytes and increased lesion CD11b^+^Ly6C^lo^ monocytes, although donor EOS did not affect lesion DC or neutrophil content (Figure 6H–J). We made similar observations in recipient mouse spleens to those from AAA lesions. Donor ILC2 from WT mice, but not those from *Il5^−/−^* mice, increased splenic EOS, but did not affect splenic Ly6C^hi^ or Ly6C^lo^ monocytes, DCs, or neutrophils. Donor EOS however, increased splenic EOS and Ly6C^lo^ monocytes and reduced splenic pro‐inflammatory Ly6C^hi^ monocytes and neutrophils (Figure S14A–D, Supporting Information).

### 2.7 Induced ILC2 Depletion Increases AAA Growth by Promoting Medial SMC Loss

**Figure 7:**
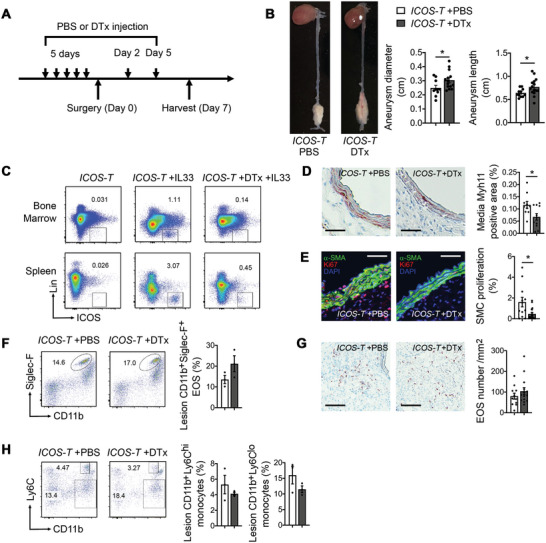
Induced ILC2 depletion exacerbates AAA growth in *ICOS‐T* mice. A) Scheme of DTx injection and surgical procedure. B) Aortic diameter and length, and representative images from *ICOS‐T* mice treated with PBS or DTx to induce ILC2 depletion. Powers: 0.560 and 0.539. C) Representative FACS analysis of bone‐marrow and splenic Lin^−^ICOS^+^ cells in *ICOS‐T* mice treated with or without IL33 for 3 d to stimulate ILC2 production, and then with or without DTx injection for 5 d to verify depletion efficiency. D) Lesion Myh11‐positive SMC areas. Scale: 25 µm. E) Immunofluorescent double staining of *α*‐actin and Ki67 to detect lesion SMC proliferation. Scale: 25 µm. FACS detected F) CD11b^+^Siglec‐F^+^ EOS, G) Siglec‐F immunostaining detected EOS (scale: 50 µm), and H) FACS detected CD11b^+^Ly6C^hi^ and CD11b^+^Ly6C^lo^ monocytes in AAA lesions from *ICOS‐T* mice with or without ILC2 depletion. Representative images for panels (B) and (D)–(H) are shown to the left. Data are mean±SEM, *n* = 12 and 15, *p < 0.05, **p < 0.01, nonparametric Mann–Whitney *U* test.

Increased AAA expansion in ILC2‐deficient *Rora^fl/fl^Il7r^Cre/+^* mice (Figure 2A) might result from reduced EOS in AAA lesions (Figure 2J), cells that are known to protect mice from AAA growth.^[^ ^11^ ^]^ Changes in AAA lesion neutrophils, DCs, and Ly6C^hi^ and Ly6C^lo^ monocytes (Figure 2K–M) may also have contributed indirectly to enhanced AAA growth in *Rora^fl/fl^Il7r^Cre/+^* mice. To test these possibilities, we used DTx to induce ILC2 depletion in *ICOS‐T* mice.^[^ ^31^, ^47^ ^]^ *ICOS‐T* mice were pretreated with DTx or phosphate‐buffered saline (PBS) for 5 d before the peri‐aortic CaPO_4_ injury (**Figure** **7**A). Induced depletion of ILC2 also increased AAA lesion diameter and length, even the calculated power did not reach to 0.80 (Figure 7B). These observations recapitulated those from the *Rora^fl/fl^Il7r^Cre/+^* mice (Figure 2A). FACS analysis revealed successful depletion of Lin^−^KLRG1^+^ICOS^+^CD127^+^ ILC2 in blood, spleen, and bone‐marrow from DTx‐treated *ICOS‐T* mice before AAA induction (Figure S15A–C, Supporting Information). Yet, DTx did not affect blood, spleen, or bone‐marrow CD3^+^CD4^+^ICOS^+^ T cells in *ICOS‐T* mice (Figure S16A–C, Supporting Information). To further ensure the efficiency of DTx‐induced ILC2 depletion, we also treated *ICOS‐T* mice with IL33 to induce ILC2 production.^[^ ^45^ ^]^ Five continuous doses of DTx were sufficient to remove ILC2 in the bone‐marrow and spleens, as assessed by FACS analysis (Figure 7C). ILC2 protected SMC from apoptosis and promoted SMC proliferation (Figure 4A–D), but ILC2 did not affect SMC collagen expression (Figure 4E) or all tested functions of ECs, DCs, or monocytes (Figure 5A–F). Consistent with these observations from in vitro assays, DTx‐induced depletion of ILC2 reduced AAA lesion SMC content as determined by myosin heavy chain 11 (Myh11) immunostaining (Figure 7D) and blunted lesion SMC proliferation as determined by immunofluorescent staining with Ki67 and *α*‐SMA antibodies (Figure 7E).

Unlike the genetic ILC2‐deficient *Rora^fl/fl^Il7r^Cre/+^* mice in which congenital ILC2 deficiency blocked ILC2‐dependent EOS development, DTx‐induced ILC2 depletion should not affect other cells within a short period after ILC2 depletion. As we expected, induced depletion of ILC2 did not affect AAA lesion EOS contents as determined by FACS analysis (CD11b^+^Siglec‐F^+^) and Siglec‐F antibody‐mediated immunostaining (Figure 7F/G). In agreement with these results, depletion of ILC2 did not affect the lesion characters that were indirectly affected by EOS deficiency in *Rora^fl/fl^Il7r^Cre/+^* mice, including lesion CD11b^+^Ly6C^hi^ or CD11b^+^Ly6C^lo^ monocyte counts (Figure 7H), CD31^+^ microvessel areas (Figure S17A, Supporting Information), and collagen I and III accumulation (Figure S17B, Supporting Information). Similar to the *Il7r^Cre/+^* and *Rora^fl/fl^Il7r^Cre/+^* mice (Figure 3A) and our previous report.^[^ ^42^ ^]^ development of AAA increased the percentage of CD11b^+^Siglec‐F^+^ EOS in spleens from *ICOS‐T* mice. ILC2‐depleted *ICOS‐T* mice showed a blunted increase of EOS (Figure S18A, Supporting Information), although blood IL5 levels did not differ between *ICOS‐T* mice treated with PBS and DTx (Figure S18B, Supporting Information).

## 3 Discussion

This study established both direct and indirect roles for ILC2 in peri‐aortic CaPO_4_ injury‐induced mouse AAA. Increase of ILC2 in AAA lesions and PaLN did not promote but rather protected mice from AAA growth. By releasing IL5, IL13, and possibly other untested molecules, ILC2 prevented SMC apoptosis and promoted SMC proliferation, suggesting a direct role for ILC2 in reducing AAA growth. By releasing IL5, ILC2 played an indirect role in preventing AAA growth by controlling EOS development. IL5‐mediated EOS development not only protected SMCs from apoptosis and promoted SMC proliferation, but also reduced endothelial adhesion molecule expression, angiogenesis, and inflammatory cell recruitment, impaired DC differentiation, and slanted monocytes from Ly6C^hi^ to Ly6C^lo^ polarization after peri‐aortic CaPO_4_ injury. ILC2 alone without EOS involvement did not exert these effects.

In addition to the genetic ILC2‐deficient *Rora^fl/fl^Il7r^Cre/+^* mice, this study used two independent approaches to test the protective role of ILC2 in AAA: ILC2 induced depletion and adoptive transfer. Adoptive transfer of in vitro prepared ILC2 from WT and *Il5^−/−^* mice allowed testing the function of ILC2‐derived IL5 in AAA. One critical question is the source of ILC2 in AAA lesions. In normal mouse aorta, we hardly detected any ILC2, but we found ILC2 accumulation in AAA lesions. Adoptively transferred donor ILC2 targeted to AAA lesions. These observations suggest that AAA‐induced ILC2 recruitment is one of the mechanisms. However, we have no evidence to exclude the possibility that resident ILC2 may expand during AAA development. Recent development of fate‐mapping mice allowed to monitor the origin and lifespan of tissue immune cells.^[^ ^48^ ^]^ *Arg1^CreERT2^* and *Id2^CreERT2^* mice have been used to monitor ILC2 de novo generation by bone‐marrow hematopoiesis and tissue local expansion. Interestingly, in most tested tissues from early adult mice, such as lung, adipose tissue, and intestine, the replenishment of residential ILC2 by de novo generated non‐fate‐mapped ILC2 population remained minimal. Early postnatal ILC2 were the major components of adult tissue resident ILC2, although ILC2 replenishment may occur at late adult life.^[^ ^49^ ^]^ These observations also support the hypothesis that the increased ILC2 in AAA lesions may be largely recruited via the chemotaxis mechanism. Therefore, use of the ILC2 fate‐mapping *Arg1^CreERT2^* and *Id2^CreERT2^* mice may give more accurate answer, which is not tested in this study.

Another limitation of our approach to test ILC2 activity in mouse AAA models is that the ILC2 from IL33‐treated mice may not behave exactly the same as those found in AAA lesions. Also, this study did not specify the roles of different ILC2 subtypes, such as IL33‐induced ILC2 (natural ILC2), IL25‐induced ILC2 (inflammatory ILC2), Thy‐1^+^Sca‐1^+^IL‐18R^+^ST2^−^C‐Kit^−^ ILC2, and CD103^+^ ILC2.^[^ ^50^ ^]^ Although it would be ideal to isolate ILC2 from AAA lesions for direct adoptive transfer, this effort is technically impossible due to the low frequency of ILC2 in AAA lesions. Therefore, prior studies used in vitro expanded ILC2 to achieve this goal even though such ILC2 lost their expression of ST2 and KLRG1.^[^ ^23^, ^24^ ^]^ Here we isolated splenic ILC2 from CD45.1 transgenic mice pretreated with IL33 for adoptive transfer studies. Use of these ILC2 avoided possible phenotypic changes from in vitro expansion and allowed monitoring and in situ characterization of donor ILC2 in AAA lesions. Immunofluorescent staining revealed the expression of ILC2 markers ST2, ICOS, CD25, PD1, IL5, and IL13 from donor ILC2 in AAA lesions. DTx‐induced ILC2 depletion is another essential approach to test ILC2 function in AAA without the impact from other AAA‐pertinent immune cells. FACS analysis showed successful depletion of ILC2 in blood, spleen, and bone‐marrow from DTx‐treated *ICOS‐T* mice, while CD3^+^CD4^+^ICOS^+^ T cells were not affected. Importantly, DTx‐induced ILC2 depletion also did not change EOS contents in AAA lesions, unlike the *Rora^fl/fl^Il7r^Cre/+^* mice in which EOS were deficient in blood, spleen, bone‐marrow and in AAA lesions. AAA lesion phenotypes in DTx‐induced *ICOS‐T* mice and *Rora^fl/fl^Il7r^Cre/+^* mice correlated with these ILC2 and EOS deficiencies and supported the hypothesis that ILC2 played a direct role in SMC apoptosis and proliferation, whereas EOS affected SMCs, ECs, DCs, and monocytes. Although ILC2 deficiency or depletion did not change the numbers of many tested immune cell types, our study did not test whether ILC2 deficiency or depletion affected the functions of these immune cells. A recent study reported in a mouse model of colonic adenocarcinoma that T‐cell subset accumulation (defined by their expression of the master control genes that regulate their development and function) in the tumor tissue did not differ between *Rora^fl/fl^Il7r^Cre/+^* and *Il7r^Cre/+^* mice, including Th1 cells (Tbet‐positive), Th2 cells (GATA3‐positive), and Treg (Foxp‐positive). Furthermore, *γδ* T cells, macrophages, DCs, mast cells, NK cells, and CD19^+^MHC‐II^+^ B‐cell migrations into the tumors were also not affected by ILC2 deficiency in *Rora^fl/fl^Il7r^Cre/+^* mice.^[^ ^51^ ^]^ These observations suggest that ILC2 deficiency or depletion did not affect the function of these cells.

ILC2‐derived IL5 or other untested molecules may affect other anti‐inflammatory cells after peri‐aortic CaPO_4_ injury, although this study did not explore these possibilities. ILC2 did not directly affect Ly6C^hi^ or Ly6C^lo^ polarization, but EOS did. As an indirect mechanism, EOS‐controlled Ly6C^hi^ to Ly6C^lo^ polarization may have contributed to increased AAA growth in ILC2‐deficient *Rora^fl/fl^Il7r^Cre/+^* mice. EOS comprise the usual target of IL5, but IL5 also affects B1 and B2 cells and controls B‐cell‐mediated innate and acquired immune responses. IL5 controls B1 cell homeostasis, proliferation, survival, and natural antibody production. It stimulates B1 cell production of prototypic IgM against ox‐LDL.^[^ ^52^, ^53^ ^]^ These antibodies block macrophage ox‐LDL uptake and foam cell formation and reduce atherogenesis. In adult mice, anti‐IL5 antibody treatment reduced B1 cell number and size.^[^ ^54^ ^]^ IL5 deficiency caused severe reduction in B1 cells in the peritoneal cavity in mouse neonates,^[^ ^55^ ^]^ and increased atherosclerosis.^[^ ^52^, ^53^ ^]^ In B2 cells, IL5 enhances anti‐CD38 antibody‐stimulated proliferation and IgG1 antibody production.^[^ ^56^ ^]^ Although a direct test of B1 cell function in AAA has not been explored, adoptive transfer of B2 cells suppresses elastase perfusion‐induced AAA in mice.^[^ ^57^ ^]^ Anti‐CD20 antibody‐mediated depletion of B1 and B2 cells reduced elastase perfusion‐induced AAA in mice concomitant with increased aorta Treg.^[^ ^6^ ^]^ Therefore, as an additional indirect mechanism, ILC2 may protect aorta from AAA formation via B1 and B2 cell mechanisms, a hypothesis that merits further exploration.

One essential question remains to be answered is whether ILC2 in human AAA lesions play similar direct and indirect roles as we claimed in mouse AAA in this study. Due to the surface marker complexity, it is technically difficult to use immunostaining, immunoblot, or ELISA to detect or quantify these cells or their molecules in human AAA lesions, although ICOS^+^GATA3^+^, ICOS^+^, or IL17RB^+^ cells have been considered tissue ILC2.^[^ ^26^, ^58^, ^59^ ^]^ Although it is also technically impractical to isolate ILC2 from human AAA lesions, study of human ILC2 in fresh blood donors from AAA patients is possible and may provide important information whether blood ILC2 contents correlate with AAA lesion size or growth rate. Moreover, surgical specimens usually sample late stage lesions that may not reflect accurately immune modulation of lesion generation.

As the first direct test of ILC2 function in AAA, this study suggests that these innate immune cells play similar protective roles in other cardiovascular diseases. We have recently reported a role of EOS in AAA and cardiac repair during myocardial infarction and cardiac hypertrophy.^[^ ^11^, ^44^, ^60^ ^]^ ILC2 may use the same mechanisms demonstrated in this study to produce IL5 to control EOS development as an indirect mechanism that protects hearts from infarct injury‐associated cardiac dysfunction.

## 4 Experimental Section

### Mice and AAA Model

C57BL/6 (000664), *Il5^−/−^* (003175), *Apoe^−/−^* (002052), and 45.1 transgenic (002014) mice aged 7–8 weeks were purchased from the Jackson laboratory (Bar Harbor, ME). *Il13^−/−^* mice were reported previously.^[^ ^61^ ^]^ The ILC2‐deficient *Rora^fl/fl^ Il7r^Cre/+^* (carrying a Rora deletion in Il7ra‐expressing lymphocytes that leads to ILC2 deficiency in the absence of neurological defects) and *Icos^fl‐DTR‐fl/+^Cd4^Cre/+^* (*ICOS‐T*, carrying a loxP‐flanked diphtheria toxin receptor (DTR) gene inserted into the *ICOS* locus, which is expressed “preferentially” on T cells and ILC2 cells, that enables *CD4^Cre^*‐mediated excision of the DTR gene from T cells and its retention in ILC2 cells, enabling ILC2 depletion through diphtheria toxin and sparing of T cells) were previously reported.^[^ ^31^ ^]^

AAA was induced by CaPO_4_ in male mice at the age of 8 to 12 weeks as described previously.^[^ ^25^ ^]^ Briefly, mice were weighed and anaesthetized with ketamine (100 mg kg^−1^), xylazine (10 mg kg^−1^), and analgesic meloxicam (3 mg kg^−1^). The abdominal aorta between the renal arteries and bifurcation of the iliac arteries was isolated from the surrounding retroperitoneal structures. A small piece of gauze soaked in 0.25 m CaCl_2_ was applied around the aorta. After 10 min, the gauze was replaced by another piece of phosphate‐buffered saline (PBS)‐soaked gauze for 5 min. Sham‐operated mice were used as experimental controls and similarly treated with 0.9% sterile saline for 15 min. To allow aorta to customize sufficient amounts of eosinophils (EOS) or ILC2 before the AAA operation, it was decided to give each recipient mouse one dose of 1 × 10^7^ donor EOS or 3 × 10^5^ donor ILC2 from WT, 45.1 transgenic or *Il5^−/−^* mice by intravenous (i.v.) injection before AAA operation. Mouse aortas were harvested at 7 d after AAA induction. AAA was also induced by Ang‐II perfusion into 8–12 weeks old male *Apoe^−/−^Rora^fl/fl^Il7r^Cre/+^* and *Apoe^−/−^Il7r^Cre/+^* mice^[^ ^11^ ^]^ and by peri‐aortic elastase injury in 8 to 12 weeks old male *Rora^fl/fl^Il7r^Cre/+^* and *Il7r^Cre/+^* control mice.^[^ ^36^, ^37^ ^]^ Anesthesia was achieved with ketamine, xylazine and meloxicam as described above. All animal procedures conformed to the Guide for the Care and Use of Laboratory Animals published by the US National Institutes of Health and was approved by the Brigham and Women's Hospital Standing Committee on Animals (protocol #2016N000442).

### Mouse Aortic Tissue Immunohistochemical and Immunofluorescent Staining

Serial cryostat cross‐ sections (6 µm) were prepared and used for immunostaining to detect CD31 (angiogenesis marker, 1:1500, 553370, BD Biosciences, Billerica, MA), CD4^+^ T cells (CD4, 1:90, 553043, BD Biosciences, Franklin Lakes, NJ), SMC (Myh11, 1:2000, 702544, Thermo Fisher Scientific, Waltham, MA), EOS (Siglec‐F, 1:50, 552125, BD Biosciences). Collagen‐I and collagen‐III deposition in lesions were determined by picrosirius red staining. Under a polarized microscope, Sirius red stained collagen I shows red–orange color and collagen III appears green. Lesion cell proliferation was assessed by immunofluorescent staining with rabbit antimouse/human Ki67 mAb (1:300, ab15580, Abcam, Cambridge, MA), followed by Alexa Fluor 555 (1:300, A‐21428, Thermo Fisher Scientific). Lesion cell apoptosis was detected by TUNEL staining (S7100, EMD Millipore, Burlington, MA). The relative CD31, SMC, and collagen contents within the aortas were quantified by measuring the immunostaining signal‐positive area using computer‐assisted image analysis software (Image‐Pro Plus; Media Cybernetics, Bethesda, MD). Lesion CD4^+^ T cells, media and adventitia Ki67‐positive proliferating cells and apoptotic cells were counted manually under the microscope.

SMC proliferation and apoptosis in mouse AAA lesions were identified by immunofluorescent double staining with FITC‐conjugated mouse anti‐*α*‐actin mAb (SMC, 1:500, F3777, Sigma‐Aldrich), together with Ki67 (1:300, ab15580, Abcam) and Alexa Fluor 594 conjugated cleaved caspase‐3 (1:100, 8172, Cell Signaling Technology, Danvers, MA). Secondary antibodies used Alexa Fluor 555 (1:300, A‐21428, Thermo Fisher Scientific).

Immunofluorescent staining was also used to detect donor ILC2 and EOS from CD45.1 transgenic mice (Cat# 002014, Jackson Laboratory). ILC2‐deficeint *Rora^fl/fl^Il7r^Cre/+^* mice received adoptive transfer of CD45.1‐positive donor ILC2 and EOS, followed by producing CaPO_4_ injury‐induced AAA. At 7 d after AAA formation, lesion sections were prepared and stained with FITC‐conjugated mouse anti‐CD45.1 mAb (1:200, 1795‐01 or 1795‐02, Southern Biotech, Birmingham, AL). Second antibody used biotin‐conjugated secondary antibodies (1:300, 0287, Sigma‐Aldrich) or Alexa Fluor 555 (1:500, A‐21422, Thermo Fisher Scientific), followed by streptavidin‐conjugated Fluor 488 (1:300, S32354, Life Technologies, Carlsbad, CA) and streptavidin‐conjugated Fluor 555 (1:500, S32355, Life Technologies). Expression of ST2, ICOS, PD1, IL5, and IL13 on CD45.1^+^ donor ILC2 in *Rora^fl/fl^Il7r^Cre/+^* recipient mouse AAA lesions was determined by immunofluorescent double staining with FITC‐conjugated mouse anti‐CD45.1 mAb (1:200, 1795‐02, Southern Biotech) together with ST2 (1:200, PA5‐20077, Invitrogen, Waltham, MA), ICOS (1:100, 313502, BioLegend, San Diego, CA), CD25 (1:50, 12‐0251‐82, Invitrogen), PD1 (1:100, PA5‐20350, Invitrogen), IL5 (1:200, PA5‐96761, Invitrogen), and IL13 (1:200, PA5‐96053, Invitrogen). Secondary antibodies used Alexa Fluor 555 (1:500, A‐21428, Thermo Fisher Scientific), Alexa Fluor 488 (1:300, 2179202, Thermo Fisher Scientific), Alexa Fluor 488 (1:300, 554026, BD Biosciences), or appropriate biotin‐conjugated secondary antibodies (1:300, 0287, Sigma‐Aldrich), followed by streptavidin conjugate Fluor 488 (1:300, 1571714, Life Technologies) and streptavidin‐conjugated Fluor 555 (1:500, 1579029, Life Technologies). Slides were counterstained with DAPI and mounted in fluorescence mounting medium. Images were collected under an Olympus FluoView FV1000 confocal microscope. Ki67‐positive and apoptotic SMCs were quantified by measuring the immunostaining Ki67‐positive and cleaved caspase‐3‐positive areas using Image‐Pro Plus software. Data were analyzed by two independent observers. Investigators were blinded to the sources of samples.

### Spleen, Aorta, or AAA Lesion, Bone‐marrow, and Blood Single Cell Preparation and Flow Cytometry

The spleen was removed from the mouse, placed in a cold PBS, and grinded in 5 mL PBS before being filtered through a 70 µm cell strainer. Splenocytes were collected after depleting the red blood cells by using the ACK Lysing buffer (A1049201, Thermo Fisher Scientific). The aortas and AAA lesions were removed, minced into small pieces, and then digested in a prewarmed 1x Aorta Dissociation Enzyme Stock Solution containing collagenase type I (450 U mL^−1^, LS004196, Worthington Biochemical Corp., Lakewood, NJ), collagenase type XI (125 U mL^−1^, C7657, Sigma‐Aldrich), hyaluronidase type I‐s (60 U mL^−1^, H3506, Sigma‐Aldrich), and deoxyribonuclease I (60 U mL^−1^, D4527, Sigma‐Aldrich) at 37 °C for 1 h. Following the incubation, single cell suspensions were prepared from the digested aortas by shearing the aortas apart and passing through a 70 µm cell strainer into 5 mL polypropylene FACS tubes (BD Falcon). Bone‐marrow cells were collected from mouse femurs and tibias, centrifuged at 300 g for 5 min. Cell suspensions were collected after lysing red blood cells using the ACK Lysing buffer. Blood was taken from the mouse heart, and the peripheral blood cells were collected after lysed red blood cells as described above.

Flow cytometry (FACS) was performed to quantify different types of immune cells in the spleen, aortas, or AAA lesions, bone‐marrow, and peripheral blood. Cells were washed and stained with the Fixable Viability Dye eFluor 450 (1:200, 65‐0863‐14, eBioscience, San Diego, CA) or Fixable Viability Dye eFluor 660 (1:200, 65‐0864‐14, eBioscience) and cell surface marker antibodies including CD45.1 (1:200, 45‐0453‐82, eBioscience), CD45 (1:200, 11‐0451‐85, eBioscience) or CD45 (1:200, 45‐0451‐82, eBioscience), CD11b (1:200, 17‐0112‐83, eBioscience), CD11c (1:200, 53‐0114‐82, eBioscience), Siglec‐F (1:200, 12‐1702‐82, eBioscience), Ly6C (1:200, 53‐5932‐82, eBioscience), Ly6G (1:200, 47‐5931‐82, eBioscience), MHC‐II (1:200, 107607, BioLegend), CD3 (1:200, 100334, BioLegend), CD4 (1:200, 11‐0042‐85, eBioscience), CD8 (1:200, 100708, BioLegend), mouse hematopoietic lineage antibody cocktail (1:40, 88‐7772‐72, eBioscience), ICOS (1:200, 25‐9942‐82, Invitrogen), KLRG1 (1:200, 46‐5893‐82, eBioscience), and CD127 (1:200, 47‐1271‐82, eBioscience). Lineage depletion was used to enrich Lin^−^ cells for ILC2 FACS analysis using the Mouse Hematopoietic Lineage eFluor 450 cocktail kit that identify, enrich, and/or deplete cells committed to the T cells, B cells, NK cells, myeloid and erythroid lineages based on the expression of their cell surface antigens (1:40, 88‐7772‐72, eBioscience). This cocktail contains the following antibodies: antimouse CD3 (17A2) eFluor 450, antimouse CD45R (B220) (RA3‐6B2) eFluor 450, antimouse CD11b (M1/70) eFluor 450, antimouse TER‐119 (TER‐119) eFluor 450, and anti‐Mouse Ly‐G6 (Gr‐1) (RB6‐8C5) eFluor 450.

To determine the different subsets of T help (Th) cells, single cell suspensions of spleen, blood, and bone‐marrow were collected and adjusted to 2 × 10^6^ cells mL^−1^. Cells were activated with phorbol myristate acetate (PMA, 50 ng mL^−1^, 16561‐29‐8, Sigma‐Aldrich) and ionomycin (1 µg mL^−1^, 10634, Sigma‐Aldrich) for 2 h followed by addition of monensin (1:1000 dilution, 420701, BioLegend) for another 3 h to evaluate Th1, Th2, and Th17 cells as reported previously.^[^ ^62^, ^63^, ^64^ ^]^ For the analysis of Treg, single cells were used directly for antibody staining. For cell surface staining, single cells were incubated with CD3 (1:200, 100222, BioLegend) and CD4 (1:200, 11‐0042‐85, eBioscience) antibodies for Th1, Th2, Th17, and Treg analysis and with CD25 antibody (1:200, 12‐0251‐82, eBioscience) for Treg analysis. For intracellular staining, cells were re‐suspended in a fixation and permeabilization solution according to the manufacturer's instructions (00‐5521‐00, Invitrogen) and then stained with IFN‐*γ* (Th1, 1:200, 12‐7311‐82, eBioscience), IL4 (Th2, 1:200, 504106, BioLegend), IL17A (Th17, 1:200, 506922, BioLegend), and Foxp3 (Treg, 1:200, 17‐5773‐82, eBioscience) respectively. Th1 cells were defined as CD3^+^CD4^+^IFN‐*γ* ^+^ cells, Th2 as CD3^+^CD4^+^IL4^+^ cells, Th17 as CD3^+^CD4^+^IL17A^+^cells, and Treg as CD4^+^CD25^+^Foxp3^+^ cells.

### IL5 ELISA

Mouse blood was collected from mouse heart, and the plasma IL5 level was determined using ELISA kit according to the manufacturers’ instructions (88‐7064‐88, Invitrogen).

### Aortic SMC Culture and Western Blot

Mouse aortic SMCs were isolated as the following steps: Briefly, mouse aortas were separated and the peri‐aortic adipose tissue was cleaned as much as could be to avoid possible contamination. The adventitia was digested with 2 mg mL^−1^ collagenase type‐II (LS004177; Worthington Biochemical Corp.) at 37 °C in water bath for 10 min. Aortas were then cut into small pieces and digested in 1 mg mL^−1^ collagenase type‐II at 37 °C for 45 min with shaking every 15 min. After digestion, 10 mL of DMEM were added containing 10% fetal bovine serum (FBS) to stop. After centrifugation, cells were resuspended with fresh media (DMEM with 10% FBS and 1% penicillin and streptomycin) and plated on a 60 mm culture dish. Media was changed every 2 d. When the SMCs became confluent, the cells were transferred to new cell culture plates. Cells will be ready to use after passage 4. SMCs were pretreated with or without EOS lysates from WT mice at 1 × 10^6^ EOS mL^−1^, ILC2 lysates from WT *Il13^−/−^* and *Il5^−/−^* mice at 2 × 10^4^ ILC2 mL^−1^, and different concentrations of mouse recombinant IL5 (10, 100, and 200 ng mL^−1^, 405‐ML, R&D Systems, Minneapolis, MN) and mouse recombinant IL13 (10 ng mL^−1^, 575904, BioLegend) for 24 h. SMCs were then incubated with 10 ng mL^−1^ TGF‐*β*, 20% FBS, and PDTC respectively. Immunoblot analysis tested the expression of p‐Smad2 (1:1000, 3108S, Cell Signaling Technology), p‐Smad3 (1:1000, ab52903, Abcam), Smad2 (1:1000, 5339S, Cell Signaling Technology), Smad3 (1:500, 9523S, Cell Signaling Technology), p‐AKT (1:1000, 9271S, Cell Signaling Technology), cleaved caspase‐3 (1:500, 9661L, Cell Signaling Technology), and GAPDH (1:3000, 2118S, Cell Signaling Technology). Immunoblots were quantified by gel density analysis using the Image J software.

### Aortic SMC Apoptosis and TUNEL Staining

Mouse aortic SMCs were plated onto an 8‐well chamber slide and pretreated with or without EOS lysates from WT mice at 1 × 10^6^ EOS mL^−1^, ILC2 lysates from WT and *Il5^−/−^* mice at 2 × 10^4^ ILC2 mL^−1^, and 10 ng mL^−1^ mouse IL5 (405‐ML, R&D Systems) for 48 h. Then SMCs were incubated with or without 100 × 10^−6^ m PDTC (P8765, Sigma‐Aldrich) for 5 h to induce apoptosis. Apoptotic SMCs were fixed with methanol for 1 min and assessed with the in‐situ apoptosis detection kit (S7100, EMD Millipore). Slides were counterstained with DAPI and mounted in fluorescence mounting medium. Images were collected under an Olympus FluoView FV1000 confocal microscope. Apoptotic SMCs were counted using ImageJ software.

### Aortic SMC Proliferation Assay

Aortic SMC proliferation was assessed using the Cell Titer 96AQ Assay kit, according to the manufacturer's instructions (G3581, Promega, Madison, WI). In brief, aortic SMCs were plated on a 96‐well plate in 100 µL DMEM with 10% FBS per well. After cells reached confluence, 100 µL of DMEM (with 10% FBS) with EOS lysates from WT mice at 1 × 10^6^ EOS mL^−1^, ILC2 lysates from WT and *Il5^−/−^* mice at 2 × 10^4^ ILC2 mL^−1^, and 10 ng mL^−1^ mouse recombinant IL5 were added to each well. After 2 d of culture, 20 µL of a mixture of tetrazolium compound and phenazine methosulfate was added into each well, and the absorbance was determined at 492 nm.

### Endothelial Cell Culture and Western Blot

Mouse aortic ECs were isolated from WT mice. Briefly, mouse aortas were removed and separated from the peri‐aortic tissues as much as possible. Aortas were minced and digested in a prewarmed collagenase‐II (2 mg mL^−1^, LS004177, Worthington Biochemical Co) buffer at 37 °C in water bath for 45 min with shaking every 15 min. Digestion was stopped by addition of double volume of the isolation medium. Digested aortic tissue was passed through a 70 µm cell strainer and washed the strainer with 15 mL of isolation media to stop the digestion. Cell suspension was centrifuged at 1800 rpm for 5 min at 4 °C. After aspirating the supernatant, cells were resuspended in 3 mL of cold PBS and incubated with PECAM‐1 (platelet endothelial cell adhesion molecule) (553389; BD Biosciences) antibody‐conjugated Dynabeads (11035; Thermo Fisher Scientific) at 7.5 µL of beads per mL of cell suspension at room temperature for 12 min. The tube containing the cells was then mounted on a magnetic separator, left for 1 min and resuspended with 0.1% bovine serum albumin (BSA) in PBS. Then the supernatant was removed, and this step was repeated for 3–5 times. Cells were re‐suspended in 10 mL of growth medium containing high glucose DMEM with 20% FBS, 1% penicillin‐streptomycin, 1% sodium pyruvate, 1% nonessential amino acids, 1% L‐glutamine, 2.5% HEPES, 1% EC growth supplement (BT‐203, Sigma‐Aldrich), and 1% heparin. Cells with beads were plated in a gelatin‐coated T25 flask. Cells were allowed 1 full day of growth, and then half of the media was changed every other day. Cells were cultured in a growth medium for next experiment. Immunoblot analysis tested the expression of ICAM‐1 (1:1000, AF796, R&D Systems), VCAM‐1 (1:1000, ab134047, Abcam), and GAPDH (1:3000, 2118S, Cell Signaling Technology). Immunoblots were quantified by gel density analysis using the Image J software.

### Aortic Ring Assay

An aortic ring assay was performed to test the role of EOS lysates and ILC2 lysates in angiogenesis. In brief, a 96‐well plate was coated with 50 µL of matrigel (354234, Discovery Labware, Inc. Bedford, MA). A 1 mm long mouse aortic ring from WT mouse was laid on the top of the solidified matrigel and covered with 100 µL of matrigel. After solidification at 37 °C cell incubator, 150 µL of RPMI (with 10% FBS) with mouse EOS lysates from WT mice at 1 × 10^6^ EOS mL^−1^, ILC2 lysates from WT and *Il5^−/−^* mice at 2 × 10^4^ ILC2 mL^−1^ were added to each well. After 7 d of culture, the aortas were photographed, and relative EC sprouting areas were analyzed using the Image‐Pro Plus software. Basic fibroblast growth factor (bFGF) (10 ng mL^−1^, D‐60240, PromoCell, Heidelberg, Germany) was used as a positive control.

### Bone‐Marrow‐Derived Monocytes Culture

Bone‐marrow‐derived monocytes were prepared from WT (C57BL/6) mice as described.^[^ ^65^ ^]^ Briefly, bone‐marrow cell suspensions were prepared by flushing femurs and tibias of 8–10‐week‐old WT mice. Cells were washed twice with medium, adjusted to give a suspension of 10^6^ cells mL^−1^, and seeded on 6‐well ultra‐low attachment surface plates (3473, Sigma‐Aldrich). Cells were supplemented with 20 ng mL^−1^ M‐CSF (315‐02, PeproTech, Rocky Hill, NJ) and cultured in a humidified incubator at 37 °C and 5% CO_2_. After 5 d, following adherent macrophages depleting, M‐CSF‐pretreated monocytes were stimulated with IFN‐*γ* (10 ng mL^−1^, 315‐05, PeproTech) in the presence of LPS (10 ng mL^−1^, L2880, Sigma‐Aldrich) for Ly6C^hi^ monocyte^[^ ^66^, ^67^ ^]^ or with IL4 alone (20 ng mL^−1^, 214‐14, PeproTech) for Ly6C^lo^ monocyte polarization, together with or without WT EOS lysates at 1 × 10^6^ EOS mL^−1^ and ILC2 lysates at 2 × 10^4^ ILC2 mL^−1^ for 48 h. Ly6C^hi^ and Ly6C^lo^ monocyte polarization was assessed by FACS analysis with CD11b (1:200, 17‐0112‐83, eBioscience) and Ly6C (1:200, 53‐5932‐82, eBioscience) antibodies.

### Bone‐Marrow‐Derived Dendritic Cell Culture

Bone‐marrow‐derived DCs were prepared from WT (C57BL/6) mice. Briefly, bone‐marrow cells were collected from femurs and tibias of 8–10‐week‐old WT mice. Cells were cultured in RPMI‐1640 medium supplemented with 10% FBS, 100 U mL^−1^ penicillin and streptomycin, IL4 (20 ng mL^−1^, 214‐14, PeproTech), and GM‐CSF (315‐02, PeproTech), adjusted to give a suspension of 0.5 × 10^6^ cells mL^−1^, and seeded on 6‐well plates. On day 3 and day 5, half of the culture medium was replaced with the medium containing IL4 and GM‐CSF. On day 7, cells were reseeded on a new 6‐well plate, and treated with or without WT EOS lysates at 1 × 10^6^ EOS mL^−1^ and ILC2 lysates at 2 × 10^4^ ILC2 mL^−1^ for 24 h. DC purity was tested by FACS analysis with CD11c (1:200, 53‐0114‐82, eBioscience) and MHC‐II (1:200, 107607, BioLegend) antibodies.

### Eosinophil Culture and Reconstitution

Mouse EOS were prepared and cultured from WT (C57BL/6) and CD45.1 transgenic mice (002014, C57BL/6, N25) as previously reported.^[^ ^11^, ^44^ ^]^ In brief, bone‐marrow cells were collected from mouse femurs and tibias, centrifuged for 5 min at 300 g, followed by lysing red blood cells using the ACK Lysing buffer (A1049201, Thermo Fisher Scientific). Cells were the suspended in 10 mL of PBS prior to cell counting. Cells with a concentration of 10^6^ cells mL^−1^ were plated in an 80 cm^2^ flask and cultured in a base media containing mouse stem cell factor (100 ng mL^−1^, 250‐03, PeproTech) and mouse Flt‐3‐ligand (100 ng mL^−1^, 250‐31L, PeproTech) for 2 d. On day 2, one‐half of the media from each flask was replaced with a fresh medium containing mouse stem cell factor and Flt‐3‐ligand. On day 4, culture media were replaced with the same media containing recombinant mouse IL5 (10 ng mL^−1^, 405‐ML, R&D Systems). Half of the culture media were changed with fresh media containing IL5 every 2 d until the 14th day. On day 14, cells were collected, and cell purity was tested by FACS analysis with CD11b (1:200, 17‐0112‐83, eBioscience) and Siglec‐F (1:200, 12‐1702‐82, eBioscience). For reconstitution, EOS were collected and counted on day 14. Each recipient mouse received one dose of 1 × 10^7^ EOS by intravenous (i.v.) injection before AAA operation. To prepare the EOS lysate for cell treatment, 1 × 10^7^ EOS were resuspended in 1 mL culture medium. After 5 cycles of freezing and thawing, cells were centrifuged at 3000 rpm. Supernatants were then collected and stored at −80 °C.

### ILC2 Isolation, Reconstitution, and Cell Lysate Preparation

Mice (WT, *Il5^−/−^, Il13^−/−^* or CD45.1 transgenic mice) aged 8–10 weeks were intraperitoneally injected with IL33 at a concentration of 500 ng per mouse for 3 d. Mouse spleens were carefully removed from these mice after perfused with 20 mL of cold PBS and grinded into single cells followed by filtering through a 70 µm cell strainer. Single cell suspension was then separated by density gradient centrifugation in a 15 mL tube layered with 5 mL lymphocyte separation medium (ICN50494, MP Biomedicals, Irvine, CA) and 5 mL cells suspension premixed with PBS. After centrifugation at 400 g for 30 min, the lymphocyte layer between lymphocyte separation medium and PBS was carefully obtained and washed with PBS and autoMACS Running Buffer (130‐091‐221, Miltenyi Biotec Inc. Cambridge, MA) twice respectively. ILC2 were then enriched using a Lineage Cell Depletion Kit (130‐090‐858, Miltenyi Biotec Inc.) according to the manufacturer's protocols. The enriched cells were then stained with cell viability dye (65‐0864‐14, eBioscience), CD45 (11‐0451‐85, eBioscience), mouse hematopoietic lineage antibody cocktail (Lin, 88‐7772‐72, eBioscience), ICOS (25‐9942‐82, eBioscience), KLRG1 (46‐5893‐82, eBioscience), and CD127 (47‐1271‐82, eBioscience) at 4 °C for 30 min. ILC2s were sorted using a FACSAria‐SORP cell sorter and suspended with PBS containing 0.1% BSA. Each recipient mouse received one dose of 3 × 10^5^ ILC2 from WT, 45.1 transgenic, or *Il5^−/−^* mice by intravenous (i.v.) injection before AAA operation. FACS detected donor CD45.1^+^ ILC2 in bone‐marrow at 1, 3, 5, and 7 d after peri‐aortic CaPO_4_ injury‐mediated AAA induction. To prepare the ILC2 lysate for cell treatment, 1 × 10^5^ ILC2 were resuspended in 1 mL culture medium. After 5 cycles of freezing and thawing, cells were centrifuged at 3000 rpm. Supernatants were then collected and stored at −80 °C.

### Statistical Analysis

All mouse data were expressed as mean±SEM. Shapiro‐Wilk test was used to determine data distribution normality. Nonparametric Mann–Whitney U test followed by least significant difference (LSD) correction was used to examine the statistical significance between two groups of abnormally distributed variables. T‐test or one‐way analysis of variance (ANOVA) test followed by LSD's post hoc correction was used for comparisons of paired parameters or multiple groups with normally distributed variables. The Kruskal–Wallis test followed by Dunn's procedure was conducted for multiple comparisons with abnormally distributed variables. Power calculations were performed for all mouse AAA models. SPSS 20.0 and Prism 8 (GraphPad) software were used for statistical analysis. *p* < 0.05 was considered statistically significant.

## Conflict of Interest

The authors declare no conflict of interest.

## Author contributions

Y.Z., T.L., Z.D., W.F., and X.Z. contributed equally to this work. Y.Z., T.L., Z.D., W.F., and X.Z. performed most of the in vivo mouse model, in vitro cell culture, and relevant analysis. M.W., J.L., S.L., G.K.S., and D.L. assisted the in vivo mouse models, immunoblots, immunohistology, and statistical analysis. A.N.J.M. provided the key reagents and critical reading of the manuscript. J.G. and G.‐P.S. participated in experimental design and data interpretation. P.L. and G.‐P.S. wrote the article. All authors contributed to the final editing and approval of the manuscript.

## Supporting information

Supporting Information

## Data Availability

The data that support the findings of this study are available from the corresponding author upon reasonable request.
