# Supplementary material for: Group 2 Innate Lymphoid Cells Protect Mice from Abdominal Aortic Aneurysm Formation via IL5 and Eosinophils
Source: Adv Sci (Weinh). 2023 Jan 2;10(7):2206958. doi: 10.1002/advs.202206958 (PMC9982556; doi:10.1002/advs.202206958)
Supplement: Supplementary file 1 — Supporting Information [file ADVS-10-2206958-s001.pdf]

# Supplemental Figures

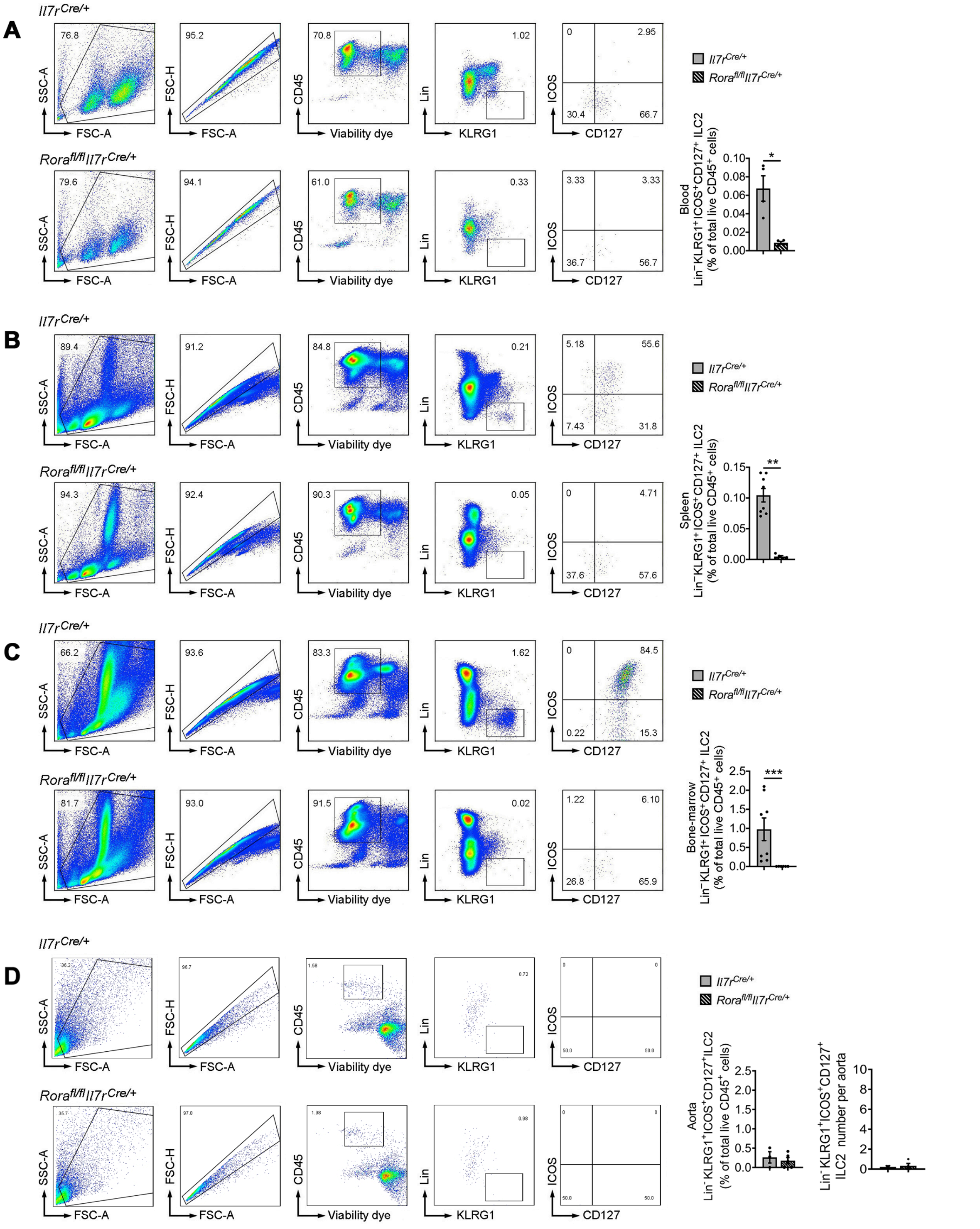

**Figure S1.** FACS analysis of Lin$^-$KLRG1$^+$ICOS$^+$CD127$^+$ ILC2 in blood (**A**), spleen (**B**), bone-marrow (**C**), and abdominal aorta (**D**) from *Il7r*$^{Cre/+}$ (n=4~8) and *Rora*$^{fl/fl}$*Il7r*$^{Cre/+}$ mice (n=4~8) without AAA. Data are mean±SEM. **P*<0.05, ***P*<0.01, ****P*<0.001, non-parametric Mann-Whitney *U* test.

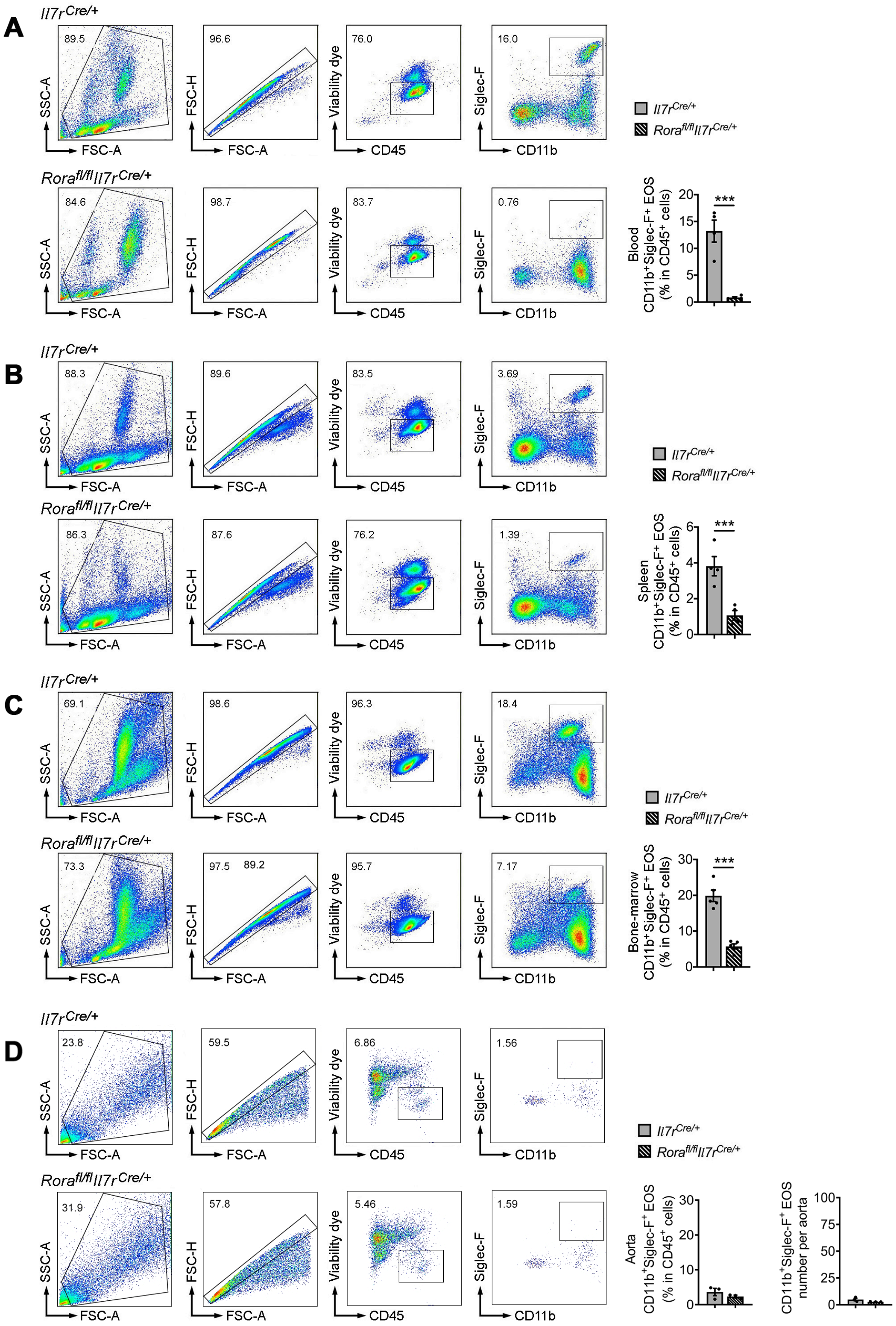


**Figure S2.** FACS analysis of CD11b$^+$Siglec-F$^+$ EOS in blood (**A**), spleen (**B**), bone-marrow (**C**), and abdominal aorta (**D**) from *Il7r*$^{Cre/+}$ (n=4~5) and *Rora*$^{fl/fl}$*Il7r*$^{Cre/+}$ mice (n=4~5) without AAA. Data are mean±SEM. ***$P$<0.001, non-parametric Mann-Whitney $U$ test.

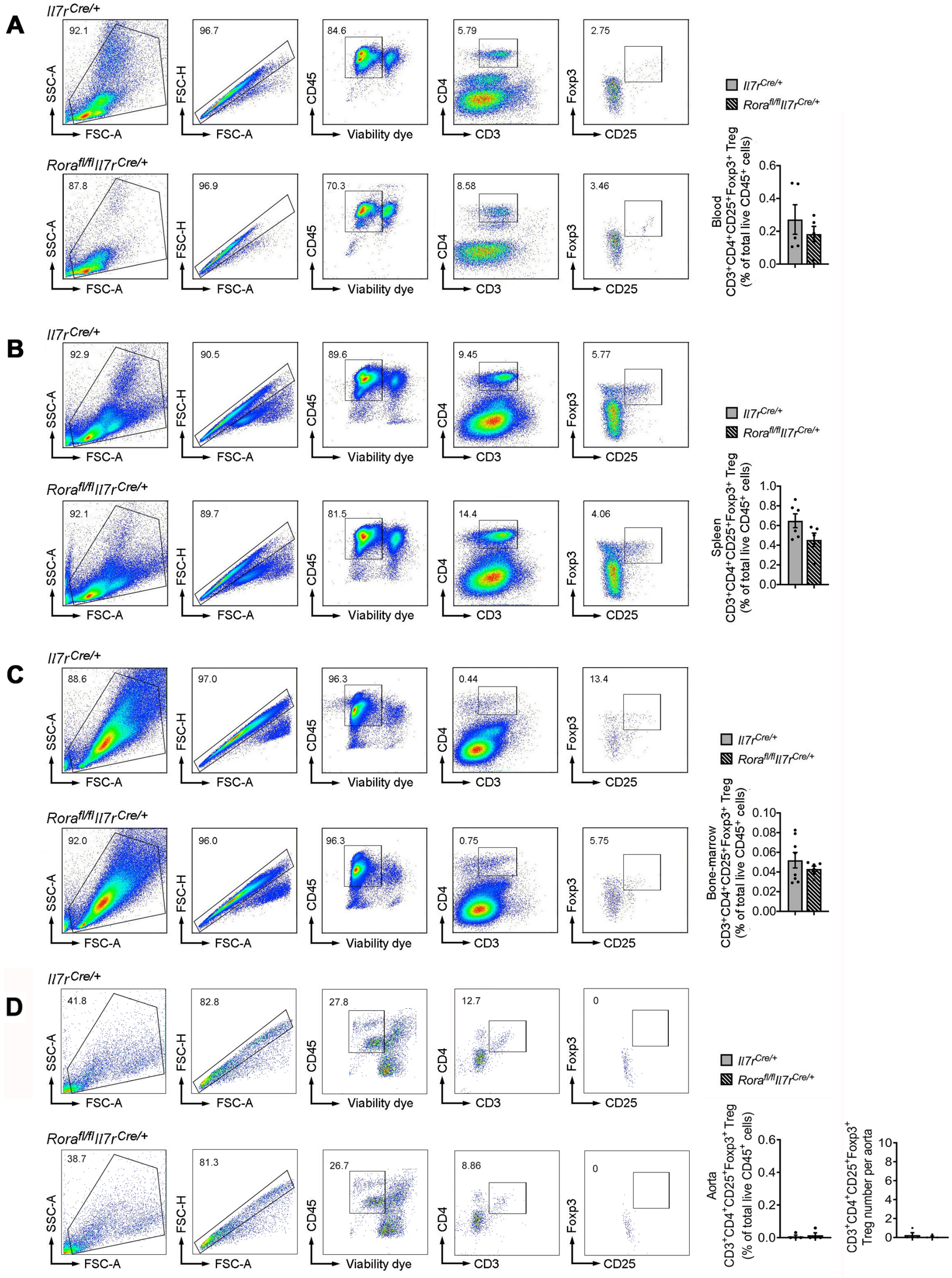


**Figure S3.** FACS analysis of $CD3^{+}CD4^{+}CD25^{+}Foxp3^{+}$ Treg in blood (**A**), spleen (**B**), bone-marrow (**C**), and abdominal aorta (**D**) from *Il7r*$^{Cre/+}$ (n=5~8) and *Rora*$^{fl/fl}$*Il7r*$^{Cre/+}$ mice (n=5~8) without AAA. Data are mean±SEM.

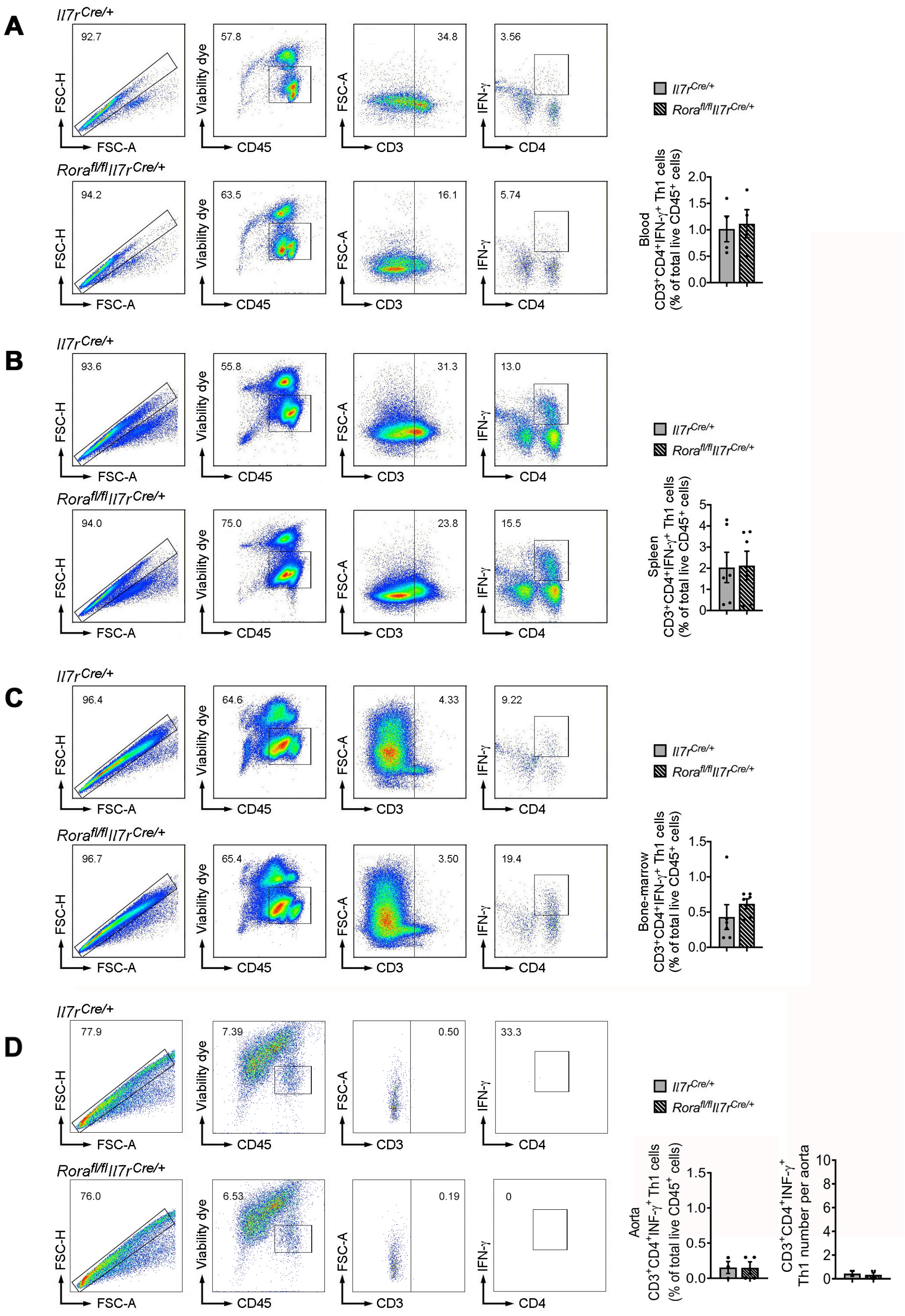


**Figure S4.** FACS analysis of CD3$^+$CD4$^+$IFN-γ$^+$ Th1 T cells in blood (**A**), spleen (**B**), bone-marrow (**C**), and abdominal aorta (**D**) from *Il7r*$^{Cre/+}$ (n=4~6) and *Rora*$^{fl/fl}$*Il7r*$^{Cre/+}$ mice (n=4~6) without AAA. Data are mean±SEM.

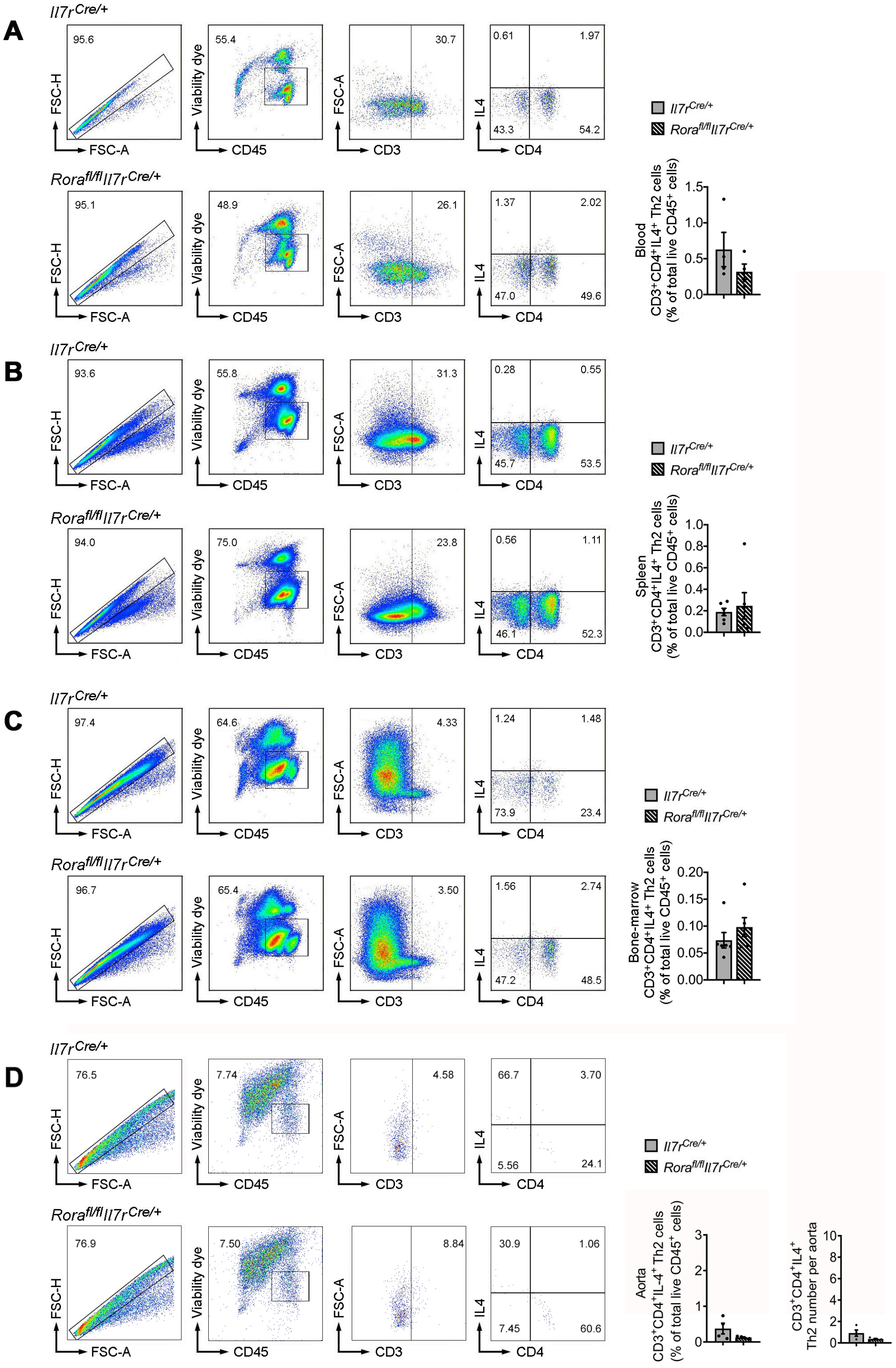


**Figure S5.** FACS analysis of $CD3^{+}CD4^{+}IL4^{+}$ Th2 T cells in blood (**A**), spleen (**B**), bone-marrow (**C**), and abdominal aorta (**D**) from *Il7r*$^{Cre/+}$ (n=4~6) and *Rora*$^{fl/fl}$*Il7r*$^{Cre/+}$ mice (n=4~6) without AAA. Data are mean±SEM.

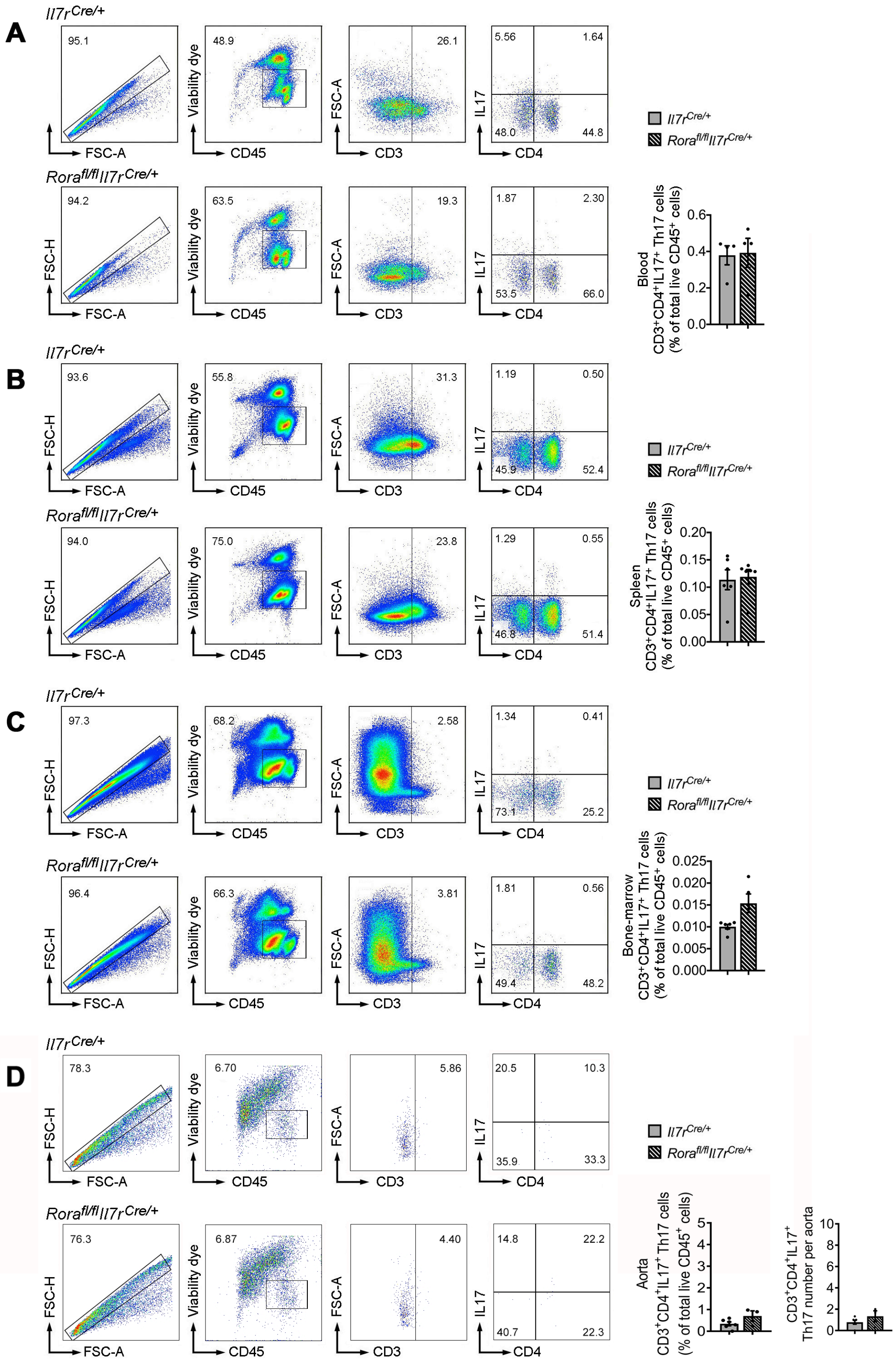


**Figure S6.** FACS analysis of $CD3^{+}CD4^{+}IL17^{+}$ Th17 T cells in blood (**A**), spleen (**B**), bone-marrow (**C**), and abdominal aorta (**D**) from *Il7r*$^{Cre/+}$ (n=4~6) and *Rora*$^{fl/fl}$*Il7r*$^{Cre/+}$ mice (n=4~6) without AAA. Data are mean±SEM.

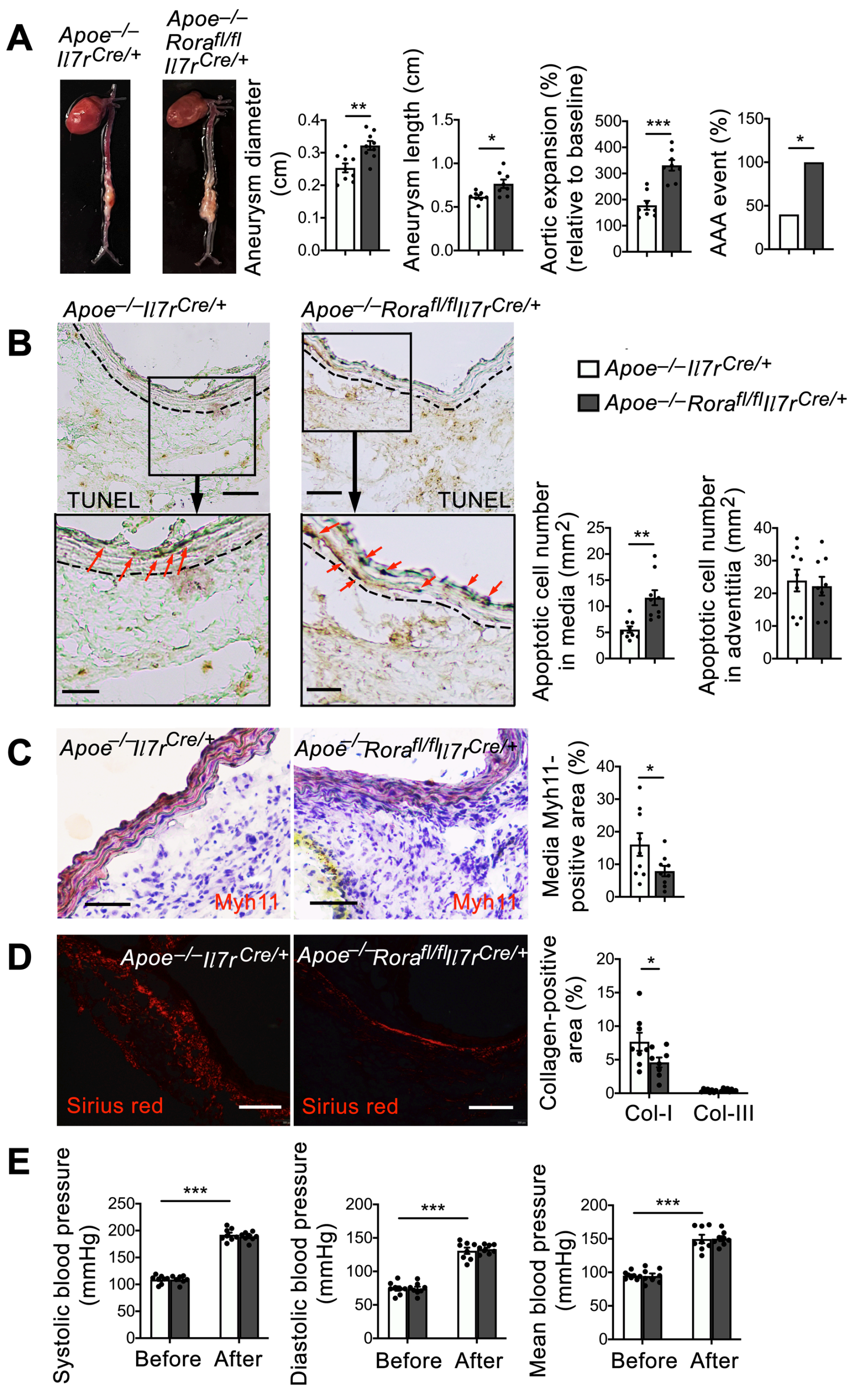


**Figure S7.** Characterization of Ang-II perfusion-induced AAA at 28 days after surgery. Both *Apoe*$^{-/-}$*Il7r*$^{Cre/+}$ (n=8) and *Apoe*$^{-/-}$*Rora*$^{fl/fl}$*Il7r*$^{Cre/+}$ mice (n=8) underwent Ang-II perfusion-induced AAA production. **A.** AAA diameter and representative images on the left, power: 0.916, AAA lesion length, Aortic diameter expansion percentage relative to the baseline (before surgery), and AAA incidence rate as defined by diameter increase by 150% or higher. **B.** TUNEL staining and media and adventitia apoptotic cell contents. **C.** Media Myh11-positive SMC area in AAA lesions. **D.** Collagen-I and –III area in AAA lesions. **E.** Blood pressures before and after AAA. Data are mean±SEM, ***P*<0.01, ****P*<0.001, non-parametric Mann-Whitney *U* test.

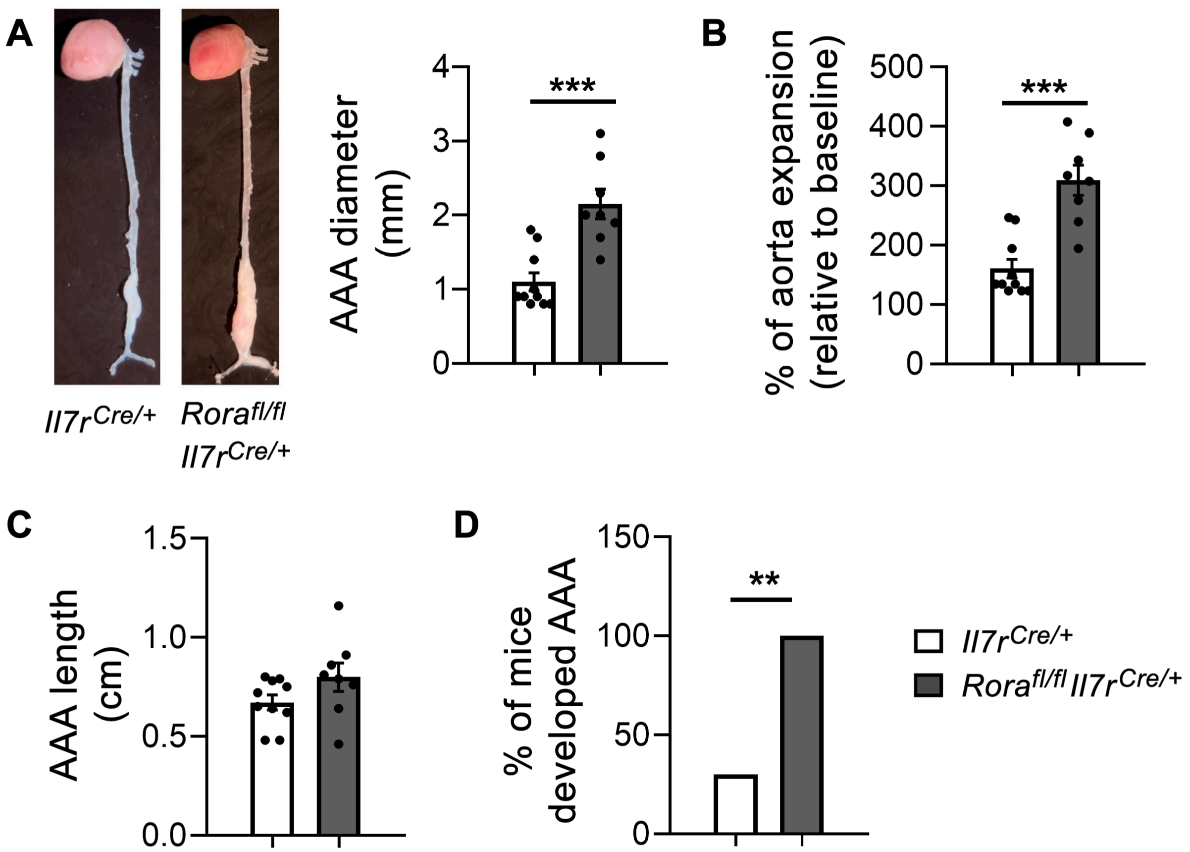


**Figure S8.** Characterization of peri-aortic elastase injury-induced AAA at 28 days after the injury. Both *Il7r*$^{Cre/+}$ (n=10) and *Rora*$^{fl/fl}$*Il7r*$^{Cre/+}$ mice (n=8) underwent peri-aortic elastase injury-induced AAA production. **A.** AAA diameter and representative images on the left. Power: 0.951. **B.** Aortic diameter expansion percentage relative to the baseline (before surgery). Power: 0.915. **C.** AAA lesion length. **D.** AAA incidence rate as defined by diameter increase by 150% or higher. Data are mean±SEM, ***P*<0.01, ****P*<0.001, non-parametric Mann-Whitney *U* test (**A-C**) and Fisher's exact test (**D**).

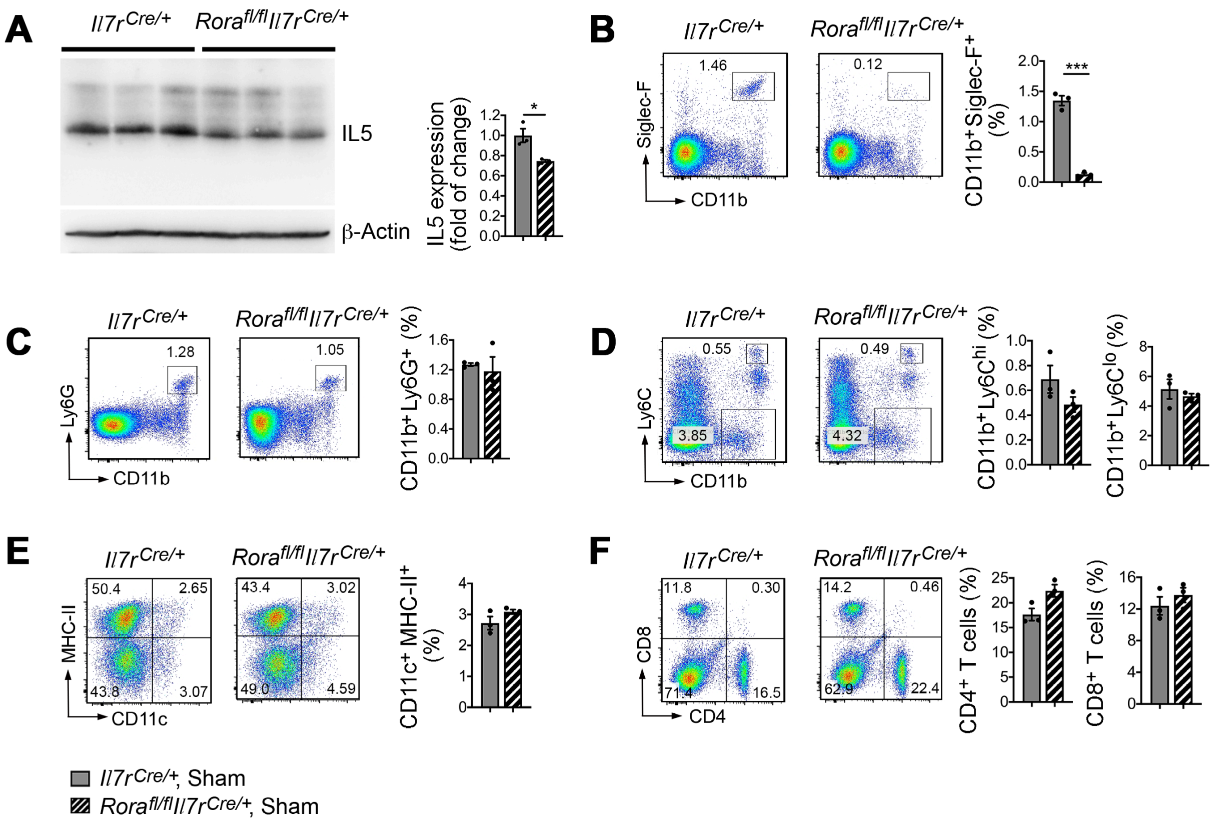


**Figure S9.** ILC2 deficiency reduces bone-marrow IL5 expression and spleen EOS contents in *Rora*$^{fl/fl}$*Il7r*$^{Cre/+}$ mice without AAA. **A.** Immunoblot analysis of IL5 in bone-marrow preparations from *Il7r*$^{Cre/+}$ and *Rora*$^{fl/fl}$*Il7r*$^{Cre/+}$ mice without AAA. n=3 each. **B-F.** FACS analysis of splenic CD11b$^{+}$Siglec-F$^{+}$ EOS (**B**), CD11b$^{+}$Ly6G$^{+}$ neutrophils (**C**), CD11b$^{+}$Ly6C$^{hi}$ and CD11b$^{+}$Ly6C$^{lo}$ monocytes (**D**), CD11c$^{+}$MHC-II$^{+}$ DCs (**E**), and CD4$^{+}$CD8$^{-}$ and CD4$^{-}$CD8$^{+}$ T cells (**F**) from *Il7r*$^{Cre/+}$ and *Rora*$^{fl/fl}$*Il7r*$^{Cre/+}$ mice before AAA formation. Data are mean±SEM. n=3 per group. *$P$<0.05, ***$P$<0.001, non-parametric Mann-Whitney *U* test.

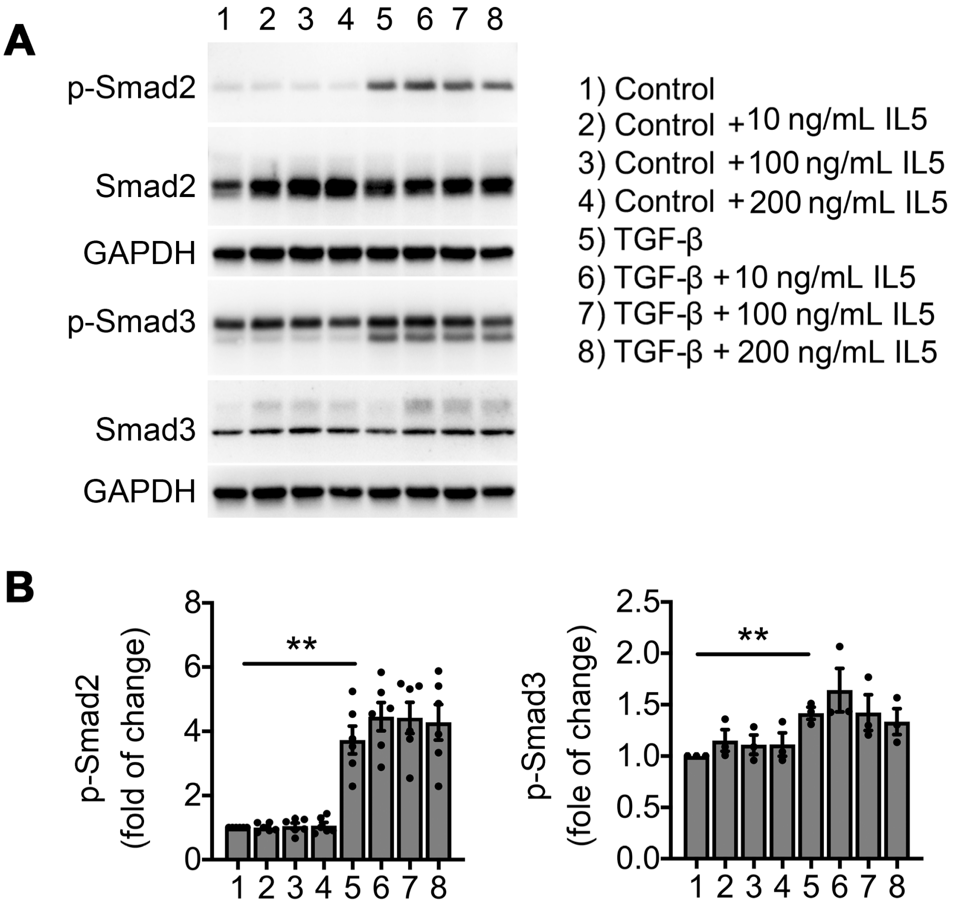


**Figure S10.** IL5 does not affect TGF-β-induced aortic SMC Smad2/3 signaling. Immunoblots (**A**) and quantification (**B**) of p-Smad2 and p-Smad3 from cultured aortic SMCs treated with or without TGF-β and different concentrations of IL5 as indicated. Data are mean±SEM. n=3~6 per group. ***P*<0.01, one-way ANOVA test.

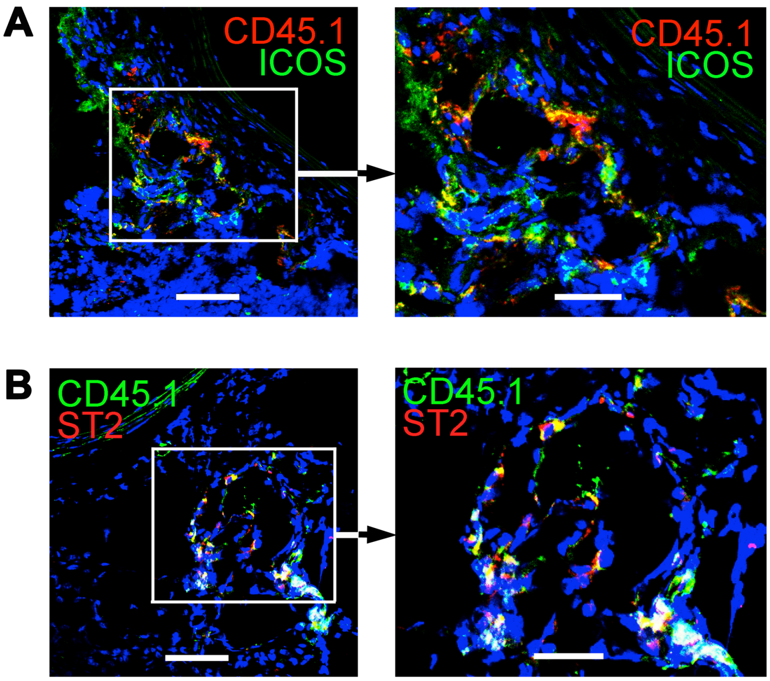


**Figure S11.** Immunofluorescent staining of AAA lesions from *Rora*$^{fl/fl}$*Il7r*$^{Cre/+}$ recipient mice received donor CD45.1$^+$ ILC2. **A.** Donor CD45.1$^+$ ILC2 expression of ICOS. **B.** Donor CD45.1$^+$ ILC2 expression of ST2.

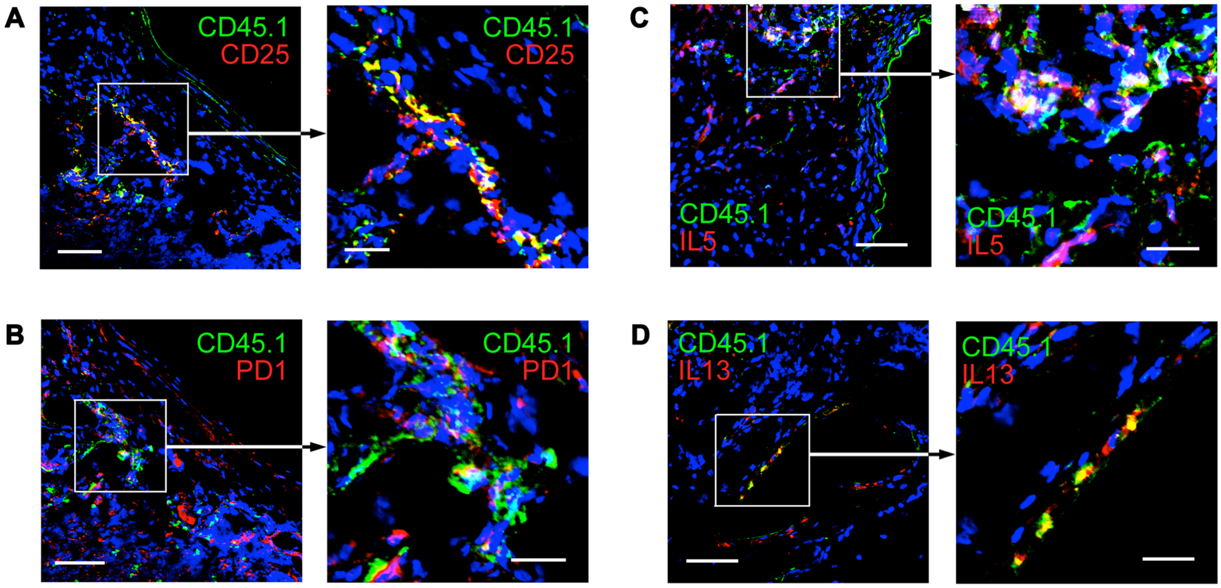


**Figure S12.** Immunofluorescent staining of AAA lesions from *Rora*$^{fl/fl}$*Il7r*$^{Cre/+}$ recipient mice received donor CD45.1$^+$ ILC2. **A.** Donor CD45.1$^+$ ILC2 expression of CD25. **B.** Donor CD45.1$^+$ ILC2 expression of PD1. **C.** Donor CD45.1$^+$ ILC2 expression of IL5. **D.** Donor CD45.1$^+$ ILC2 expression of IL13.

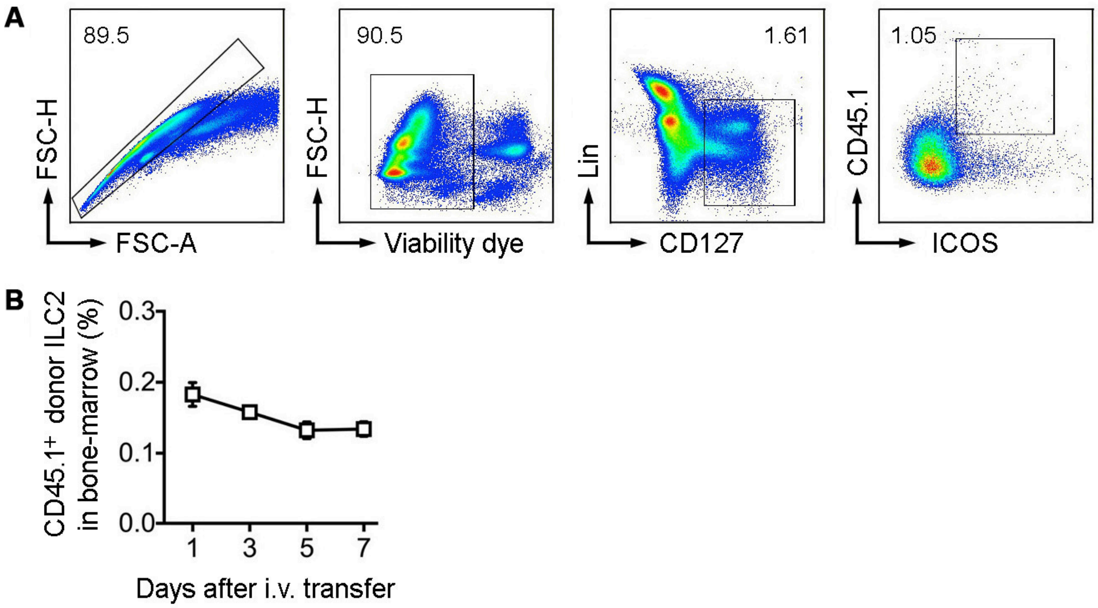


**Figure S13.** FACS analysis of bone-marrow $Lin^{-}CD127^{+}ICOS^{+}CD45.1^{+}$ donor ILC2 in *Rora*$^{fl/fl}$*Il7r*$^{Cre/+}$ recipient mice at 1, 3, 5, and 7 days after donor cell i.v. transfer and peri-aortic $CaPO_4$ injury-induced AAA. **A.** FACS gating strategy. **B.** Bone-marrow ILC2 contents. N=4 per data point.

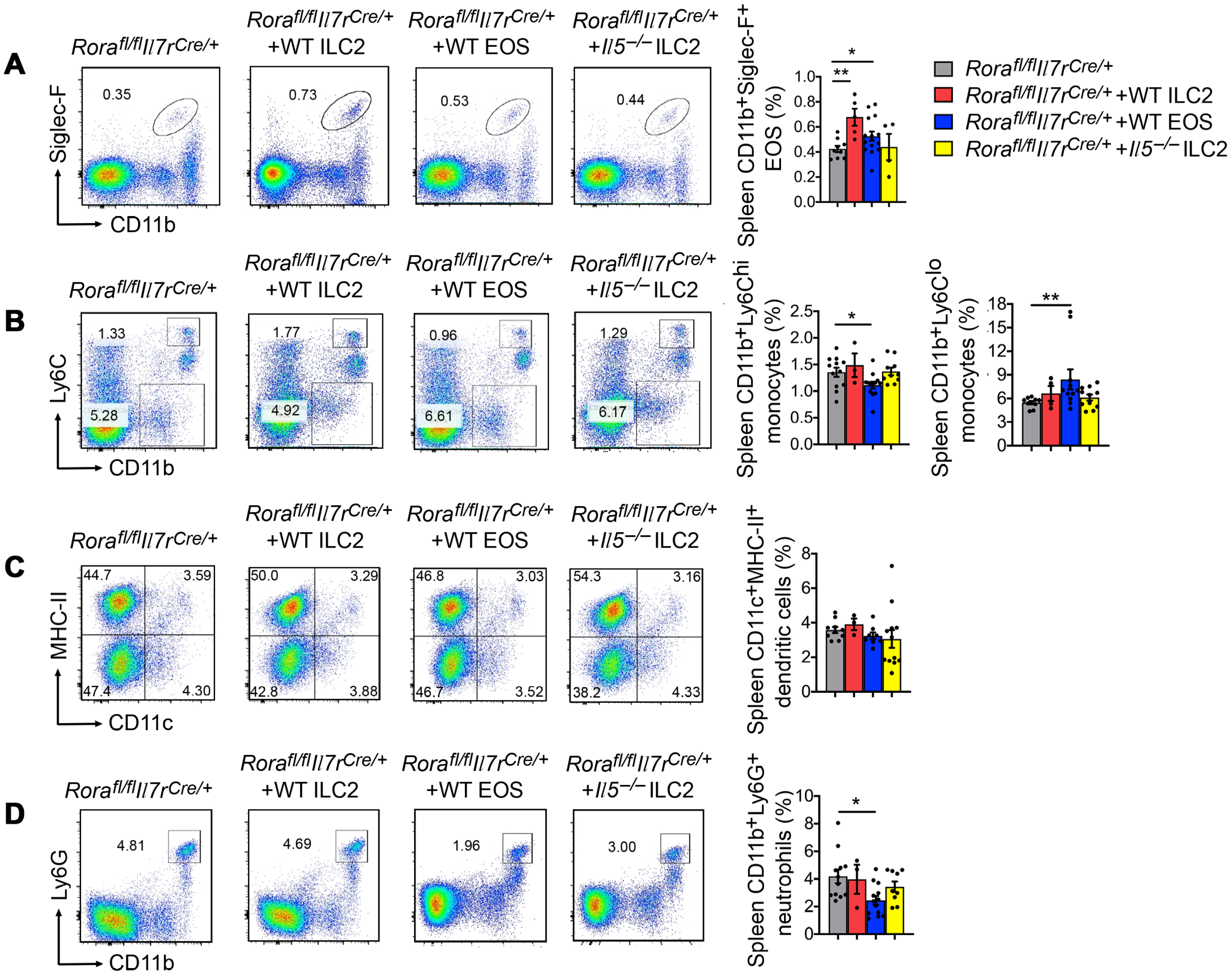


**Figure S14.** FACS analysis of splenic CD11b$^{+}$Siglec-F$^{+}$ EOS (**A**), CD11b$^{+}$Ly6C$^{hi}$ and CD11b$^{+}$Ly6C$^{lo}$ monocytes (**B**), CD11c$^{+}$MHC-II$^{+}$ dendritic cells (**C**), and CD11b$^{+}$Ly6G$^{+}$ neutrophils (**D**) from *Rora*$^{fl/fl}$*Il7r*$^{Cre}$ mice reconstituted with or without EOS from WT mice or ILC2 from WT or *Il5*$^{-/-}$ mice. Representative FACS images are shown to the left. Data are mean±SEM. n=3~15 mice per group. **P*<0.05, ***P*<0.01, one-way ANOVA test.

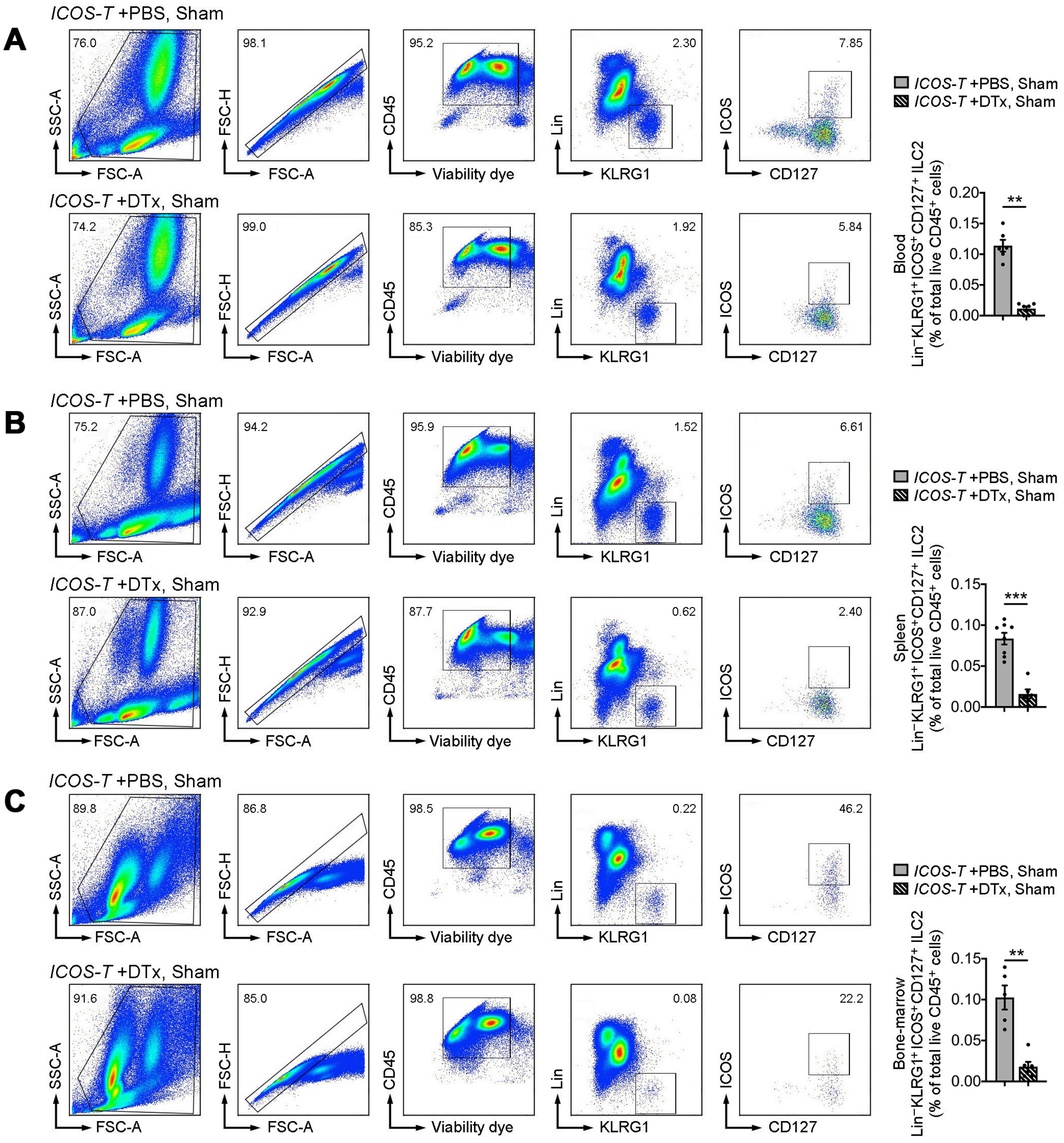

**Figure S15.** FACS analysis of Lin$^-$KLRG1$^+$ICOS$^+$CD127$^+$ ILC2. *ICOS-T* mice received PBS (n=5~8) or DTx (n=6) for 5 days, followed by FASC analysis of Lin$^-$KLRG1$^+$ICOS$^+$CD127$^+$ ILC2 in peripheral blood (**A**), spleen (**B**), and bone-marrow (**C**) in these mice without AAA production. **$P$<0.01, ***$P$<0.001, non-parametric Mann-Whitney *U* test.

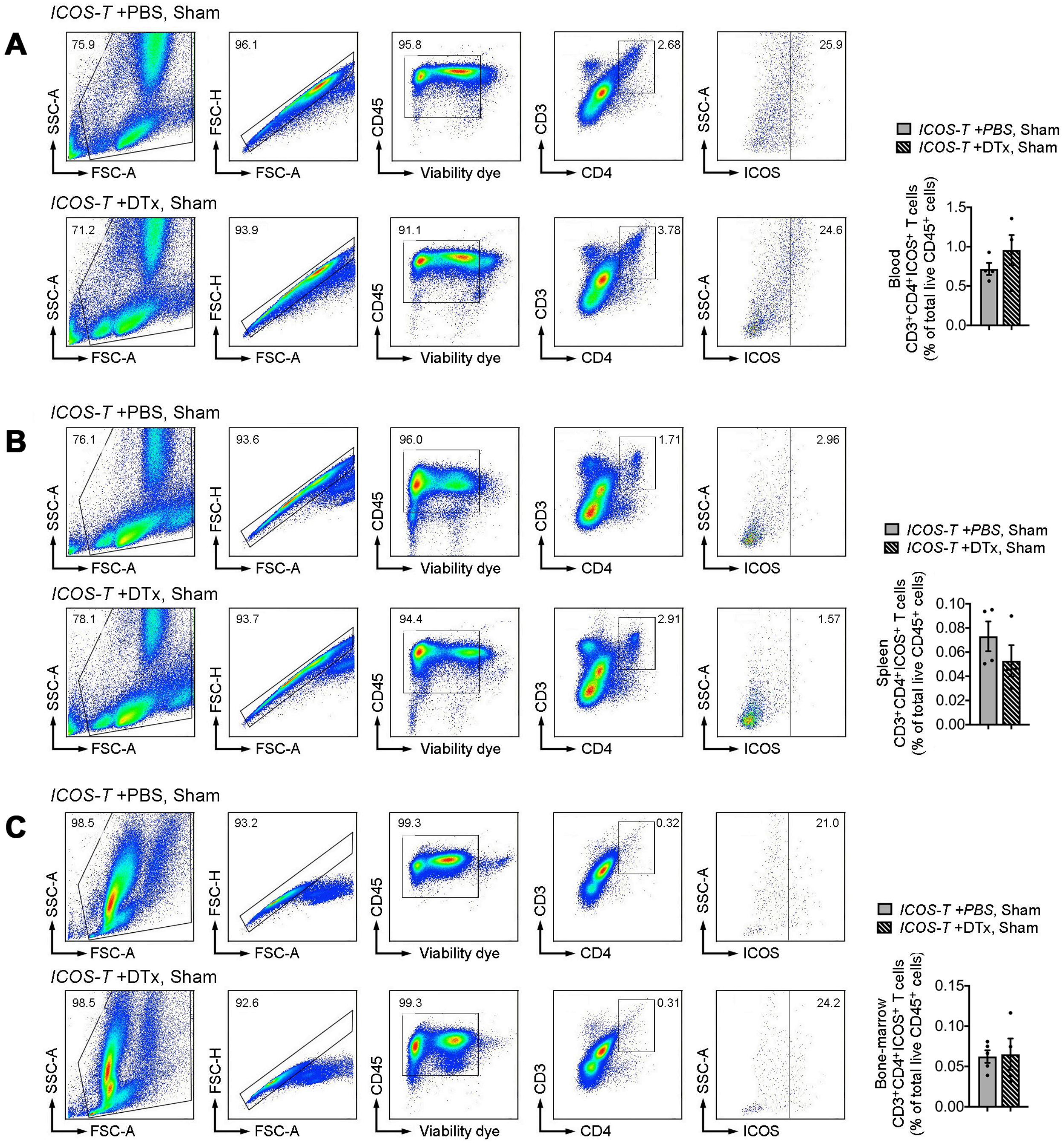


**Figure S16.** FACS analysis of CD3$^{+}$CD4$^{+}$ICOS$^{+}$ T cells. *ICOS-T* mice received PBS (n=4~5) or DTx (n=4) for 5 days, followed by FASC analysis of CD3$^{+}$CD4$^{+}$ICOS$^{+}$ T cells in peripheral blood (**A**), spleen (**B**), and bone-marrow (**C**) in these mice without AAA production.

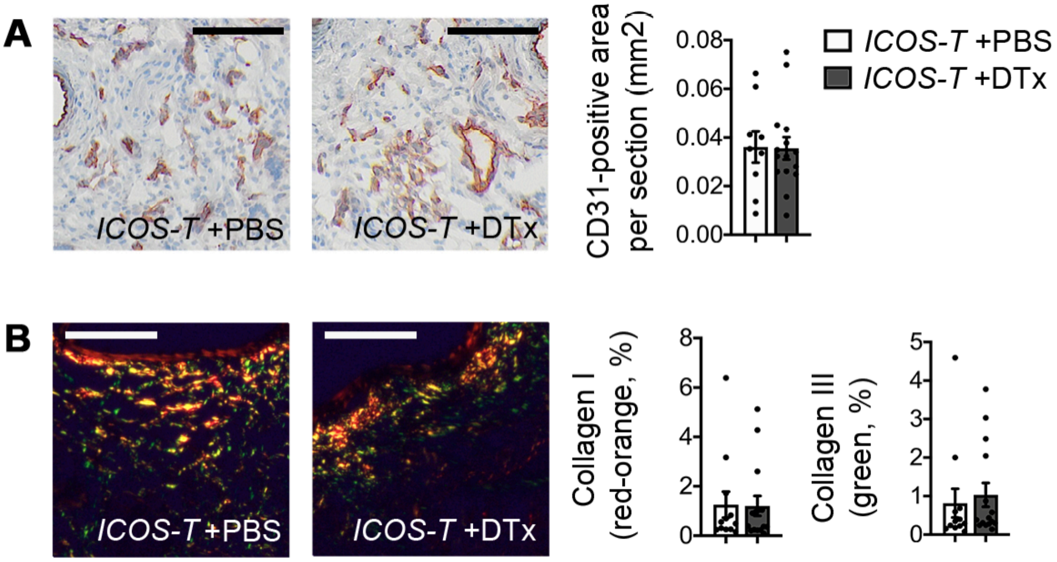


**Figure S17.** Induced ILC2 depletion did not affect AAA lesion angiogenesis or collagen deposition. **A.** Immunostaining detected CD31-positive microvessel areas in AAA lesions from *ICOS-T* mice treated with PBS or DTx. Scale: 50 μm. **B**. Sirius red staining of AAA lesions from *ICOS-T* mice treated with PBS or DTx. Scale: 50 μm. Data are mean±SEM. n=12 and 15 mice per group. Non-parametric Mann-Whitney *U* test was used.

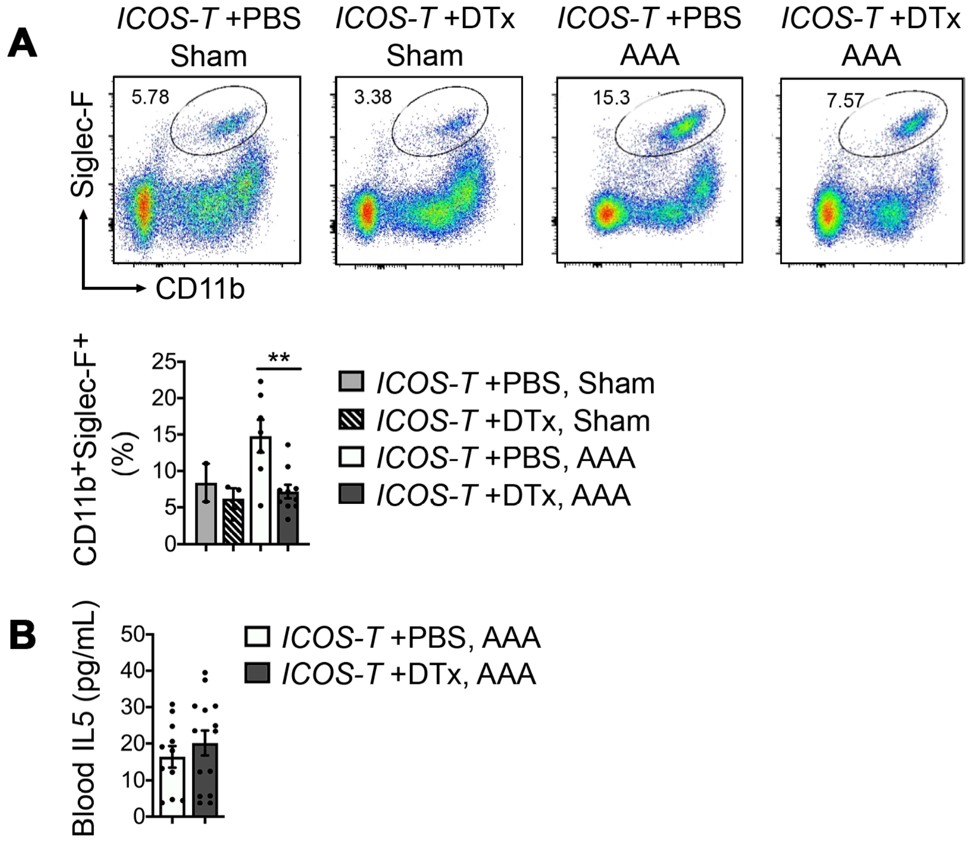


**Figure S18.** Splenic EOS and blood IL5 in *ICOS-T* mice treated with PBS or DTx. **A.** FACS analysis of splenic $CD11b^{+}Siglec\text{-}F^{+}$ EOS in *ICOS-T* mice treated with PBS or DTx. Representative FACS images were shown to the top. Data are mean±SEM. n=3~10 mice per group. ***P*<0.01, one-way ANOVA test. **B**. Blood IL5 levels in *ICOS-T* mice treated with PBS or DTx. Data are mean±SEM. n=11~14 mice per group. Non-parametric Mann-Whitney *U* test was used.
